# Thermally Stable Organic Field‐Effect Transistors Based on Asymmetric BTBT Derivatives for High Performance Solar‐Blind Photodetectors

**DOI:** 10.1002/advs.202106085

**Published:** 2022-02-19

**Authors:** Yicai Dong, Yanan Sun, Jie Liu, Xiaosong Shi, Haiyang Li, Jing Zhang, Chunlei Li, Yuanping Yi, Song Mo, Lin Fan, Lang Jiang

**Affiliations:** ^1^ Beijing National Laboratory for Molecular Sciences Key Laboratory of Organic Solids Institute of Chemistry Chinese Academy of Sciences Beijing 100190 China; ^2^ University of the Chinese Academy of Sciences Beijing 100049 China; ^3^ Key Laboratory of Science and Technology on High‐tech Polymer Materials Chinese Academy of Sciences Institute of Chemistry Chinese Academy of Sciences Beijing 100190 China

**Keywords:** asymmetric molecules, high detectivity, organic field‐effect transistors, solar‐blind photodetectors, thermal stability

## Abstract

High‐performance solar‐blind photodetectors are widely studied due to their unique significance in military and industrial applications. Yet the rational molecular design for materials to possess strong absorption in solar‐blind region is rarely addressed. Here, an organic solar‐blind photodetector is reported by designing a novel asymmetric molecule integrated strong solar‐blind absorption with high charge transport property. Such alkyl substituted [1]benzothieno[3,2‐b][1]‐benzothiophene (BTBT) derivatives C*n*‐BTBTN (*n* = 6, 8, and 10) can be easily assembled into 2D molecular crystals and perform high mobility up to 3.28 cm^2^ V^−1^s^−1^, which is two orders of magnitude higher than the non‐substituted core BTBTN. C*n*‐BTBTNs also exhibit dramatically higher thermal stability than the symmetric alkyl substituted C8‐BTBT. Moreover, C10‐BTBTN films with the highest mobility and strongest solar‐blind absorption among the C*n*‐BTBTNs are applied for solar‐blind photodetectors, which reveal record‐high photosensitivity and detectivity up to 1.60 × 10^7^ and 7.70 × 10^14^ Jones. Photodetector arrays and flexible devices are also successfully fabricated. The design strategy can provide guidelines for developing materials featuring high thermal stability and stimulating such materials in solar‐blind photodetector application.

## Graphical abstract

Asymmetric [1]benzothieno[3,2‐b][1]‐benzothiophene (BTBT) derivatives of C*n*‐BTBTNs that integrate strong solar‐blind absorption with high mobility are designed and synthesized. C10‐BTBTN‐based solar‐blind photodetectors demonstrate preeminent performance with the *P*, *R*, and D* up to 1.60 × 10^7^, 8.40 × 10^3^ A W^−1^, and 7.70 × 10^14^ Jones, respectively, which exhibit a broad prospect in optoelectronic device applications.

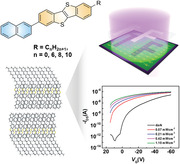

## 1 Introduction

UV communications featured by low eavesdropping and non‐line of sight communication, are highly desired for short‐distance and confidential communication.^[^ ^1^ ^]^ In the UV spectrum, wave band of 200–280 nm (solar‐blind range) solar irradiation cannot reach the earth due to strong absorption by the atmosphere, that contributes to super high signal‐to‐noise ratio.^[^ ^2^ ^]^ These features endow the solar‐blind photodetectors great significance in civil and military area including the missile warning, fire alarms, and environmental monitoring.^[^ ^3^, ^4^, ^5^ ^]^ Compared to photodiodes, phototransistors based on organic, inorganic, and organic–inorganic hybrid semiconductor materials demonstrate unique advantages of low noise, high sensitivity, and signal‐amplification function,^[^ ^6^, ^7^, ^8^, ^9^, ^10^, ^11^, ^12^ ^]^ which have been drawing great research interest in solar‐blind photodetectors.^[^ ^13^, ^14^, ^15^, ^16^, ^17^ ^]^ Among them, organic semiconductors, which possess low cost, mechanical flexibility, controllable molecular structure, and tunable optoelectronic properties,^[^ ^18^, ^19^, ^20^, ^21^, ^22^, ^23^ ^]^ would render potential applications in flexible and wearable photo response electronic devices.^[^ ^24^, ^25^, ^26^, ^27^, ^28^, ^29^ ^]^ Recently, significant improvements have been achieved in high performance organic solar‐blind photodetectors, either by interface engineering or by developing novel device architectures, and so on.^[^ ^15^, ^30^, ^31^, ^32^, ^33^ ^]^

Although much progress has been made, the vigorous development and application of high performance organic solar‐blind photodetectors still faces many challenges, such as the lack of highly responsive organic semiconductors. To obtain high performance solar‐blind photodetectors, the semiconductors should not only have strong absorption in the solar‐blind range as well as wide band gap, but also have high charge carrier mobility. The photocurrent across a phototransistor can be given by *I*_ph_ = *enμEW*, where *e, n, μ, E, and W* are the electronic charge, density of photo‐induced carriers per unit area, carrier mobility, applied electric field, and width of the device, respectively,^[^ ^6^ ^]^ so high mobility is beneficial for obtaining high photocurrent. However, the rational molecular design for materials combining strong absorption in solar‐blind region and high mobility remains blank. Novel molecular skeletons which fulfill above characteristics would boom the field of organic solar‐blind photodetectors and their applications.

[1]Benzothieno[3,2‐b][1]‐benzothiophene (BTBT) derivatives, as one of the most widely studied semiconductors in outstanding organic field‐effect transistors (OFETs),^[^ ^34^, ^35^, ^36^, ^37^, ^38^ ^]^ are of great potential in solar‐blind photodetectors due to their wide band gaps (*E*_g_) and high mobility. Indeed, the star molecule C8‐BTBT was reported with high mobility and excellent solar‐blind photo response performance, and was expected to boom the organic solar‐blind photodetectors,^[^ ^7^, ^39^, ^40^ ^]^ yet, the poor thermal stability of C8‐BTBT limits its in‐depth applications in photodetectors. Therefore, organic solar‐blind semiconductors with high thermal stability and strong absorption in the solar‐blind range are highly desired.^[^ ^41^, ^42^ ^]^

Compared with symmetric BTBT derivative, the asymmetric BTBTs that show wider band gaps^[^ ^43^, ^44^ ^]^ and comparable mobility have advantages in addressing the above challenges. Here, we report a series of novel asymmetric BTBT derivatives, named C*n*‐BTBTN (*n* = 0, 6, 8, and 10). Compared with BTBTN, molecules with alkyl chain substituents can be easily assembled into two‐dimensional (2D) molecular crystals with bilayer thickness and exhibit higher mobility. Bottom‐gate top‐contact (BGTC) single‐crystal OFETs of C6‐, C8‐, and C10‐BTBTN exhibit a maximum mobility up to 2.10, 3.28, and 2.39 cm^2^ V^−1^s^−1^, respectively, which are superior to that of BTBTN (0.10 cm^2^ V^−1^s^−1^). Meanwhile, all of them show excellent thermal stability. Besides the higher sublimation and phase transition temperatures of the C*n*‐BTBTNs (*n* = 6, 8, and 10) than C8‐BTBT, the thin film transistors (TFTs) of C10‐BTBTN also exhibit better thermal durability. Furthermore, the materials indeed show intense absorption toward the solar‐blind wavelength, and typical photodetectors based on C10‐BTBTN exhibits outstanding solar‐blind light response properties with the photosensitivity (*P*), photoresponsivity (*R*), and detectivity (D*) of 1.60 × 10^7^, 8.40 × 10^3^ A W^−1^, and 7.70 × 10^14^ Jones, respectively. Photodetector arrays for high‐resolution imaging and flexible detectors have been also successfully demonstrated.

## 2 Results and Discussions

**Scheme 1:**
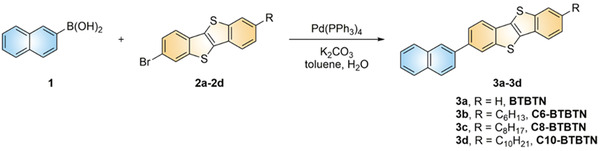
Synthetic route to BTBTN derivatives.

In order to obtain high‐performance solar‐blind light‐sensitive materials, the BTBT moiety with a fused ring system that guarantees good crystallization and charge transport property was selected as the core. The asymmetric substitution strategy was adopted to compromise the requirements of wide‐bandgap and high charge carrier mobility, and an aromatic substitution is introduced to one end of BTBT to improve the thermal stability. A series of new asymmetric BTBT structures with different aromatic substituents (named BTBTN, BTBTTB, BTBTTT, and BTBTT, respectively) were constructed and theoretical calculations were carried out to evaluate their energy levels. As shown in Figure S1, Supporting Information, the naphthyl substituted BTBT derivative (BTBTN) shows the widest bandgap and was focused for further study. The alkyl chains are attached to the other side of BTBT core to enhance the self‐assembly capability of the molecules for higher OFET performance. The synthetic route of the investigated four compounds C*n*‐BTBTN (*n* = 0, 6, 8, and 10) is presented in **Scheme** **1** and Scheme S1, Supporting Information. All of them can be easily synthesized via a simple Suzuki coupling reaction in the presence of Pd (0)‐catalyst and K_2_CO_3_, which start from the same material, naphthalene‐2‐boronic acid, and the same core structure (Br‐BTBT) with different alkyl substituents. Final products as white powders were successfully obtained in good yield, and were characterized by ^1^H NMR (Figure S2, Supporting Information) and EI‐MS and elemental analysis (see more details in Supporting Information).

**Figure 1:**
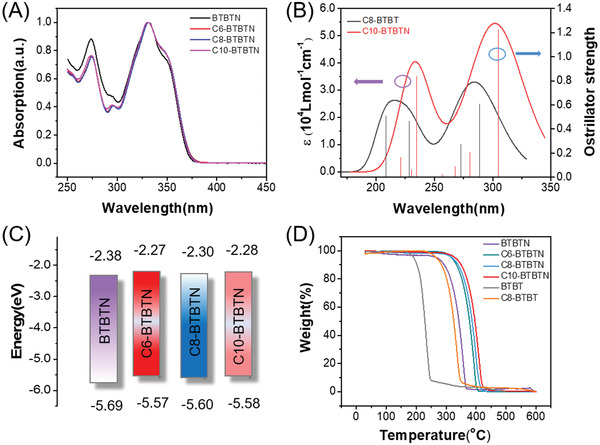
A) UV–vis absorption spectra of four compounds in 10^−5^ m dichloromethane solution. B) UV–vis absorption spectra and oscillator strengths of C10‐BTBTN and C8‐BTBT obtained by simulation calculation. C) Frontier orbital level diagrams. D) TGA results of C*n*‐BTBTN (*n* = 0, 6, 8, and 10), BTBT, and C8‐BTBT.

UV–vis and cyclic voltammetry measurements were carried out to clarify their physicochemical properties (**Figure** **1**A; Figure S3, Supporting Information). UV–vis absorption spectra of C*n*‐BTBTN (*n* = 0, 6, 8, and 10) demonstrated similar onset absorption wavelength of 375 nm, and wide band gaps of 3.30 eV were obtained by adopting the equation of *E*_g_ = 1240/*λ*, which makes them potential materials for solar‐blind photodetectors. Besides, theoretical calculations were conducted on monomers of C10‐BTBTN and the star molecule C8‐BTBT to evaluate their oscillator strengths and UV–vis absorption (in Figure 1B; Table S1, Supporting Information). The obviously enhanced absorption intensity of C10‐BTBTN than C8‐BTBT might be owing to the stronger oscillator strengths. The introduction of naphthalene ring might contribute to the increased *π*–π* transition probability of valence electron, that promote the absorption in 200–280 nm region, further confirming the potential of C10‐BTBTN for solar‐blind photodetectors.

The highest occupied molecular orbital (HOMO) energy levels were estimated from the onsets of the oxidation peaks and summarized in Figure 1C according to the equation of *E*_HOMO_ = −(4.40 + *E*_onset_) eV. The almost identical band gaps and HOMO levels of the four compounds illustrated that alkyl‐substituents have little influence in their energy levels, which coincident with theoretical calculation in Figure S4, Supporting Information that the electrons mainly located in the naphthyl and BTBT core rather than alkyl chains.

The thermal properties of the four compounds, BTBT, and C8‐BTBT were evaluated by thermogravimetric analysis (TGA) and differential scanning calorimetry (DSC). In Figure 1D, we can see that all of C*n*‐BTBTN (*n* = 0, 6, 8, and 10) compounds show good thermal stability and their sublimation temperature increase from 273 °C to 318, 319, and 329 °C with the increase of alkyl chain length. However, the sublimation temperature of BTBT and C8‐BTBT are 194 and 269 °C, respectively, which are lower than that of C*n*‐BTBTN. Besides, similar phase transition behavior of C6‐, C8‐, and C10‐BTBTN can be observed with two exothermic and endothermic peaks in heating and cooling process in the second scan in Figure S5, Supporting Information. The liquid‐crystal phase transition and melting transition temperatures are 216 and 231 °C, 206 and 226 °C, and 192 and 223 °C for C6‐, C8‐, and C10‐BTBTN, respectively. This trend is highly related to the apparent thermal motion of alkyl chains with long length.^[^ ^45^ ^]^ In comparison, C8‐BTBT has a phase transition behavior only at 109 and 125 °C. Therefore, compared to BTBT and C8‐BTBT, C*n*‐BTBTN compounds exhibited apparently improved thermal stability.

**Figure 2:**
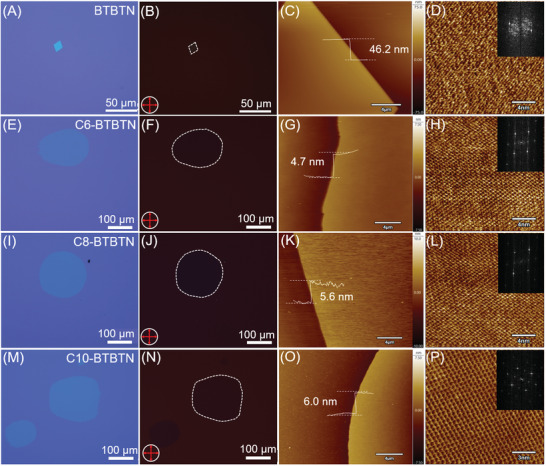
Optical microscopy images for A,E,I,M) C*n*‐BTBTN (*n* = 0, 6, 8, and 10) single crystals and B,F,J,N) corresponding POM images. AFM images and HR‐AFM images of C,D) BTBTN (the inset is corresponding 2D FFT pattern); G,H) C6‐BTBTN; K,L) C8‐BTBTN; O,P) C10‐BTBTN.

To investigate their charge transport performance, high‐quality single crystals of C*n*‐BTBTN (*n* = 0, 6, 8, and 10) were successfully obtained by physical vapor transport in argon atmosphere on OTS (octadecyltrichlorosilane)‐treated SiO_2_/Si substrates. As shown in **Figure** **2**A, the lateral size of regular quadrilateral BTBTN single crystals was about 20–30 µm with the thickness of typical crystal of 46.2 nm. While, C*n*‐BTBTN (*n* = 6, 8, and 10) prone to assemble into ultrathin molecular crystals with lateral size of 100–400 µm as shown in Figure 2 and Figure S6B–D, Supporting Information. It is concluded that asymmetric alkylation of BTBT backbone promoted large‐area 2D growth and produced crystals with obviously reduced thickness. Polarized optical microscopy (POM) images of C*n*‐BTBTN (*n* = 0, 6, 8, and 10) in Figure 2B,F,J,N prove their single crystal nature. Furthermore, the smallest thickness of C6‐, C8‐, and C10‐BTBTN were verified to be 4.7, 5.6, and 6.0 nm by atomic force microscopy (AFM) as shown in Figure 2G,K,O, which were roughly equivalent to the thickness of two molecular layers (Figure S7, Supporting Information). The obviously different growth behaviors of BTBTN and alkylated BTBTN demonstrates that the alkyl chain substitution weakens the interlayer interactions by forming bilayer herringbone structure, and assists the crystal growth along the lateral direction. The layer‐by‐layer growth behaviors with a step of bilayer are also confirmed by the multilayer crystals in Figure S8, Supporting Information.

To gain insight into in‐plane molecular arrangement and packing structure of C*n*‐BTBTN (*n* = 6, 8, and 10), typical high‐resolution AFM (HR‐AFM) characterizations and their corresponding 2D fast Fourier transform (FFT) patterns were carried out. As shown in Figure 2D,H,L,P, lattice parameters calculated from 2D FFT pattern were *a* = 5.3 Å, *c* = 8.3 Å for BTBTN, *a* = 5.6 Å, *c* = 8.3 Å for C6‐BTBTN, *a* = 5.7 Å, *c* = 8.5 Å for C8‐BTBTN, and *a* = 6.2 Å, *c* = 8.0 Å for C10‐BTBTN, respectively. The lattice parameters of C10‐BTBTN were well consistent with that of single crystals (*a* = 5.9 Å, *c* = 7.7 Å). In addition, the crystalline properties were further confirmed by out‐of‐plane X‐ray diffraction (XRD) measurements (Figure S9, Supporting Information) of C*n*‐BTBTN single crystals. The smooth baseline and sharp diffraction peaks demonstrated good crystallinity of C*n*‐BTBTN crystals.

**Figure 3:**
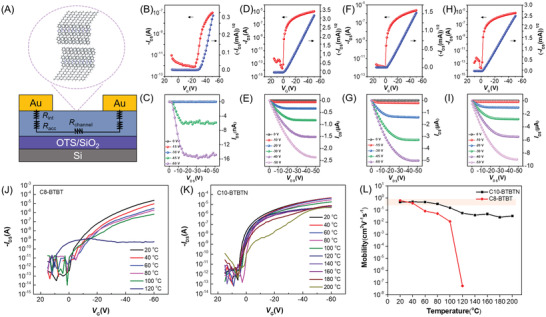
A) Schematic cross sections of BGTC OFETs. Typical transfer and output curves of B,C) BTBTN, D,E) C6‐BTBTN, F,G) C8‐BTBTN, and H,I) C10‐BTBTN devices. J,K) Typical transfer characteristics of C8‐BTBT and C10‐BTBTN TFTs at elevated temperature in steps of 20 °C. L) Corresponding mobility as a function of texting temperature.

Single‐crystal transistors were successfully fabricated with BGTC configuration by mechanical transferring gold films as source and drain electrodes,^[^ ^46^ ^]^ as shown in Figure S10, Supporting Information. Typical transfer and output measurements were performed to assess the electrical characteristics of C*n*‐BTBTN‐based devices, as shown in **Figure** **3**. In Figure 3H, the saturation mobility of C10‐BTBTN device is extracted as 1.08 cm^2^ V^−1^s^−1^ according to Equation S1, Experimental Section, Supporting Information, and the device showed a high on/off ratio of 10^7^ as well as small threshold voltage of −0.05 V. Similar good performances were also obtained for C6‐BTBTN and C8‐BTBTN (Figure 3D,F). Mobility distribution are attached in Figure S11, Supporting Information, and the average and maximum mobility of C6‐, C8‐, and C10‐BTBTN are 1.14 ± 0.44 and 2.10 cm^2^ V^−1^s^−1^, 1.90 ± 0.50 and 3.28 cm^2^ V^−1^s^−1^, and 1.47 ± 0.48 and 2.39 cm^2^ V^−1^s^−1^, respectively. As a contrast, BTBTN single‐crystal transistors exhibited poor performance with average mobility of 0.048 ± 0.023 cm^2^ V^−1^s^−1^ and maximum mobility of 0.10 cm^2^ V^−1^s^−1^. Besides the largely improved mobility, the good linearity in output curves of C*n*‐BTBTN (*n* = 6, 8, and 10) at low *V*_DS_ compared with that of BTBTN (Figure 3C) indicates low contact resistance in these bilayer crystals based OFETs (Figure S12–S14, Supporting Information). The large contact resistance in Figure 3C may be caused by the access resistance (*R*_acc_) deduced from the bulk thickness of BTBTN crystals. It is concluded that C*n*‐BTBTN semiconductors with long alkyl chains not only favor the formation of ultrathin molecular crystals but also possess improved OFET performance.

To evaluate the thermal stability of devices, C10‐BTBTN and C8‐BTBT TFTs were fabricated and tested under the continuous heating process. The transfer curves at different temperatures are shown in Figure 3J,K, and the corresponding mobility are extracted in Figure 3L. The mobility of C10‐BTBTN TFTs could preserve 70% of the initial value when tested at 80 °C and gate‐modulated charge transport behavior could be even observed when tested at 160 °C with mobility of 0.05 cm^2^ V^−1^s^−1^. However, the performance of the C8‐BTBT device shows a rapid decline when upon heating, and dropped to 50% of its pristine value only at 40 °C, then sharply decreased to 0.05 cm^2^ V^−1^s^−1^ at 80 °C, and almost lost the gate‐modulation property because of the melting of C8‐BTBT thin films when tested at 120 °C (as shown in Figure S15, Supporting Information). The largely improved thermal stability of C10‐BTBTN over C8‐BTBT indicates its great potential in thermally durable device applications.

**Figure 4:**
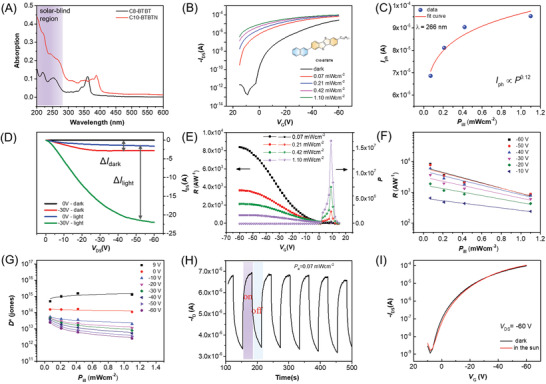
A) UV–vis absorption spectra of 30 nm C10‐BTBTN and C8‐BTBT thin films on quartz. B) Transfer characteristics of C10‐BTBTN‐based photodetector measured under 266 nm laser at different illumination intensities. C) Photocurrent as a function of illumination intensity. D) Typical output curves in the dark and under illumination. E) *P* and *R* as a function of illumination intensity. F,G) *R* and D* of the photodetectors as a function of power intensity at different *V*_DS_. H) Photoswitching characteristics. I) Transfer curves of photodetectors in the dark and in the sunlight.

According to electrical and UV–vis spectral characterizations (Figure S16, Supporting Information), it is concluded that C*n*‐BTBTN (*n* = 6, 8, and 10) achieved the integration of strong absorption in solar‐blind region with high mobility. Considering that light response performance is also influenced by interfacial defects and crystallinity of active layer,^[^ ^26^, ^47^ ^]^ TFTs were selected to fabricate photodetectors. Among the C*n*‐BTBTNs, C10‐BTBTN TFTs exhibited higher mobility and stronger absorption in solar‐blind region (Figure S16–S18, Table S2, Supporting Information). In addition to that, UV–vis absorption of C10‐BTBTN thin films is much stronger than C8‐BTBT in the solar‐blind region (**Figure** **4**A). The results indicated a great significance of C10‐BTBTN molecule in solar‐blind photodetectors. The transfer characteristics of the photodetectors in dark and under 266 nm illumination are presented in Figure 4B. In this measurement, *V*_G_ is swept from 20 to −60 V and *V*_DS_ is fixed at −60 V. During the 266 nm illumination, obviously increased *I*_DS_ and positively shifted *V*_th_ are observed with the increase of light intensity, suggesting that the C10‐BTBTN photoactive layer can generate carriers efficiently by absorbing photons. Figure 4C further illustrates the positive correlation between photocurrent and light intensity. The slower increase rate of photocurrent under stronger light intensity might be correlated to the gradually occupied surface traps. Figure 4D shows the typical output curves of the photodetector in the dark and under illumination.

Moreover, the photodetection characteristics are evaluated systematically. Figure 4E presents the *P* and *R* as a function of *V*_G_ under different light intensities. The *P* value calculated by

(1) $$P=\Delta I/I_{\mathrm{dark}}\;=\left(I_{\mathrm{ph}}-I_{\mathrm{dark}}\right)/I_{\mathrm{dark}}$$

It should be noted that *P* value in the off‐state is higher than that in the on‐state owing to positive voltage shift of *V*_th_ under illumination, and the maximum *P* for each light intensity is observed at the *V*_G_ of about 8 V. *P* is positively correlated to incident light intensity and reached a maximum of 1.60 × 10^7^ at 1.10 mW cm^−2^, which is the highest *P* value of organic solar‐blind photodetector by far, as shown in **Table** **1**. In spite of the weak light intensity of 70 µW cm^−2^, *P* still shows a high value of 3.90 × 10^5^. The outstanding *P* value of C10‐BTBTN TFTs could be attributed to the electron traps and built‐in electric field that induced more holes at dielectric/semiconductor interfaces.

**Table 1:** Representative solar‐blind photodetectors based on organic semiconductors

Materials	Wavelength [nm]	*P*	*R* [A W^−1^]	D* [Jones]	Reference
P3HT‐PHA (nanofibrils)	254	‐	120	4.2 × 10^14^	^[^ ^32^ ^]^
C8‐BTBT (ribbons)	280	8200	44	‐	^[^ ^39^ ^]^
P‐MSB (2D) (single crystal)	254	‐	2.74 × 10^4^	7.8 × 10^13^	
Perylene(2D) (single crystal)	254	‐	1.16 × 10^3^	4.54 × 10^12^	^[^ ^15^ ^]^
*α*‐6T(2D) (single crystal)	254	‐	1.29 × 10^4^	6.9 × 10^12^	
diF‐TEG ADT (thin films)	254	5.5	0.139	‐	^[^ ^48^ ^]^
OPTM (blend films)	254	1.8 × 10^4^	9.4 × 10^2^	‐	^[^ ^49^ ^]^
PVK/NSN	275	‐	0.039	‐	^[^ ^33^ ^]^
C10‐BTBTN (thin films)	266	1.60 × 10^7^	8.40 × 10^3^	7.70 × 10^14^	This work

In addition, *R* indicates the efficiency of photodetectors responding to light and is defined as

(2) $$R=\Delta I/\left(P_{\mathrm{ill}}S\right)=\left(I_{\mathrm{ph}}-I_{\mathrm{dark}}\right)/\left(P_{\mathrm{ill}}S\right)$$

where *P*_ill_ is light intensity, and *S* is channel area of the photodetectors. It is known that *R* increases with the increase of *V*_G_ at a fixed light intensity because of the increased light current. Furthermore, *R* as a function of light intensity was extracted and shown in Figure 4F. *R* decreases with the increase of light intensity at a fixed voltage. When *V*_G_ = −60 V and the light power is 70 µW cm^−2^, *R* reaches a maximum value of 8.40 × 10^3^ A W^−1^, which is much larger than those of many reported solar‐blind photodetectors (Table 1). Moreover, typical transfer and output characteristics of this device are attached in Figure S19, Supporting Information and high mobility of 0.48 cm^2^ V^−1^s^−1^ was extracted. Thus high *R*, which is gate‐/light‐tunable are successfully achieved by adopting C10‐BTBTN TFTs.

D* is also an important factor of photodetectors, and D* as a function of light intensity with various voltage are shown in Figure 4G. It was concluded by the equation of

(3) $$D^{*}=RS^{1/2}/{\left(2eI_{\mathrm{dark}}\right)}^{1/2}$$

D* slightly decreases with the increase of light intensity when applied a negative voltage that is consistent with the photocurrent change. A superhigh detectivity of 7.70 × 10^14^ Jones was achieved that can be attributed to low dark current and high responsivity (Figure S20, Supporting Information).

The photoswitching characteristics of the photodetectors were studied at a fixed *V*_G_ of −5 V under a 70 µW cm^−2^ illumination (Figure 4H). When the illumination was periodically applied or removed, the device could be easily and reversibly switched between on and off states with good stability and repeatability. What's more, Figure 4I shows the current *I*_DS_ at various *V*_G_ in dark and in sunlight. No obvious response of the photodetectors under the sun is observed and excellent solar‐blind selectivity was verified. The high photo response characteristics ensure the significant applications of C10‐BTBTN in solar‐blind photodetectors. In addition, the photo response performance of single crystal‐based photodetectors was also investigated. The transfer characteristics of single crystal‐based photodetectors in dark and under 1.10 mW cm^−2^ 266 nm illumination are shown in Figure S21, Supporting Information. The *P*, *R*, and D* were calculated as 5.38 × 10^4^, 3.67 A W^−1^, and 8.67 × 10^12^ Jones, respectively, that were lower than TFT counterparts. Further confirming the better photodetector performance of TFTs over their single crystal counterparts.^[^ ^26^ ^]^

**Figure 5:**
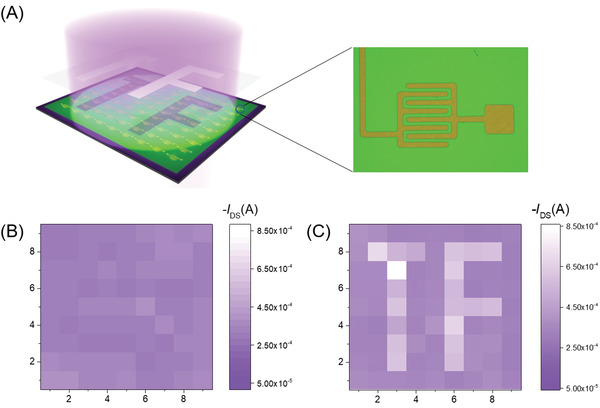
A) Schematic illustration of the photodetector array and typical device partially masked and exposed to 254 nm light. B,C) Image of current mapping of the 9 × 9 matrix tested in the dark and under 254 nm illumination of commercial hand lamp.

To explore the ability of image‐information recording, an image photodetector matrix with 9 × 9 pixels was fabricated. Schematic illustration is shown in **Figure** **5**A. In Figure 5B, the current at *V*_G_ = −60 V measured in dark was exhibited, that indicates the uniform device performance. As illustrated in Figure 5C, the photodetector array was illuminated by accessible 254 nm commercial hand lamp and the current mapping image was successfully produced by projecting a “TF” (the abbreviation of “thin film”) optical pattern onto C10‐BTBTN array. The devices and typical transfer characteristics in dark and under 254 nm illumination are attached in Figure S22, Supporting Information. Channel current in exposed area was about 4–8 × 10^−4^ A in contrast to those of the unexposed pixels (1 × 10^−4^ A), illustrating the feasibility for high resolution imaging and potential application on cameras, fax machines, and so forth.

**Figure 6:**
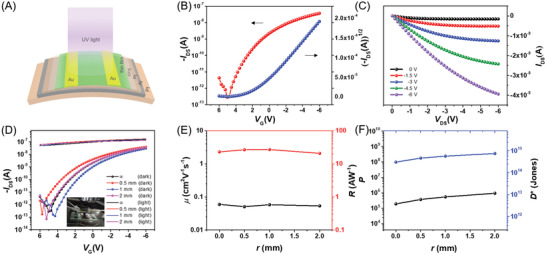
A) Schematic illustration of the C10‐BTBTN‐based flexible photodetectors. B,C) Typical transfer and output characteristics on PI substrate. D) Transfer characteristics with various bending radii in darkness and under 266 nm illumination; the inset shows the testing in bending state. E,F) Evolution of *µ*, *R*, *P*, and D* with *r*.

To extend the applications of solar‐blind photodetectors on the flexible electronics, we successfully fabricated devices on PI (polyimide) flexible substrates using an Al gate electrode and a PI dielectric. In the flexible device, low contact resistance and low voltage operation are achieved as shown in **Figure** **6**B,C. Furthermore, mechanical flexibility measurements were conducted with different curvature by bending the devices. In Figure 6D, initially, the bending radius of the curvature (*r*) of flat devices was ∞. Transfer curves of flexible OFETs in dark and under illumination shown against the different bending radii (*r* = ∞, 0.5 mm, 1mm, and 2 mm) exhibited no considerable difference except slightly‐shifted threshold voltage. The results also reflect sensitive light response performance of flexible devices, and output characteristics are shown in Figure S23, Supporting Information. The evolution of *µ*, *R*, *P*, and D* with respect to the bending radius is shown in Figure 6E,F. This flexibility test of the device with different bending radius exhibited similar performance even when bent at radii of 2 mm, suggesting the high quality of the semiconductor layer and excellent mechanical compatibility.

## 3 Conclusion

In summary, a class of novel molecules integrated strong absorption in solar‐blind region with high mobility were designed and synthesized via the asymmetric substitution of BTBT. The asymmetric naphthalene substitution promised the wide bandgap and strong solar‐blind absorption of the C*n*‐BTBTNs, and the asymmetric alkyl assisted the assembly of the molecules in the intralayer direction for ultra‐thin crystals and improved their FET performance. OFETs based on above C*n*‐BTBTN (*n* = 6, 8, and 10) single crystals devices exhibited a maximum mobility of 2.10, 3.28, and 2.39 cm^2^ V^−1^s^−1^, respectively, which are much higher than BTBTN (0.1 cm^2^ V^−1^s^−1^). Moreover, the asymmetric aromatic substitution also improved thermal stability of C*n*‐BTBTNs over C8‐BTBT, which indicates broader application scenarios of C*n*‐BTBTN devices.

Based on the strong solar‐blind absorption of C*n*‐BTBTNs, high performance solar‐blind photodetectors are achieved for C10‐BTBTN TFTs. Record‐high *P* and D* up to 1.60 × 10^7^ and 7.70 × 10^14^ Jones were achieved with *R* of 8.40 × 10^3^ A W^−1^ under 266 nm illumination. The outstanding solar‐blind photodetector performance is highly related to the strong absorption, high mobility, as well as their thin film device architecture. Photodetector arrays composed of 9 × 9 pixels were also fabricated and high‐resolution imaging of “TF” was successfully obtained. Moreover, flexible devices on PI substrate exhibited great mechanical flexibility nearly without performance degradation when bended with different radii of curvature were realized. Researches on these molecules will provide a new molecular design strategy to develop high performance solar‐blind optoelectronic materials in organic electronics.

## 4 Experimental Section

### Statistical Analysis

C*n*‐BTBTN (*n* = 0, 6, 8, and 10) single crystals could be obtained with similar results for more than 40 times independent experiment. More than 40 OFETs based on C*n*‐BTBTN (*n* = 0, 6, 8,10) single crystals were prepared and analyzed. Average mobility of BTBTN, C6‐BTBTN, C8‐BTBTN, and C10‐BTBTN single‐crystal devices were 0.048 ± 0.023, 1.14 ± 0.44, 1.90 ± 0.50, and 1.47 ± 0.48 cm^2^ V^−1^s^−1^, respectively. More than 20 OFETs based on C*n*‐BTBTN (*n* = 6, 8, and 10) thin films were prepared and analyzed. Average mobility of C6‐BTBTN, C8‐BTBTN, and C10‐BTBTN thin‐film devices were 0.14 ± 0.01, 0.13 ± 0.01 and 0.51 ± 0.14 cm^2^ V^−1^s^−1^, respectively. More than five devices of C10‐BTBTN and C8‐BTBT for thermal stability measurement were performed and showed similar thermal stability characteristics. More than five solar‐blind photodetectors based on C10‐BTBTN were fabricated and measured, and the *P*, *R*, and D* value of all of photodetectors were similar with the same order of magnitude. More than five flexible devices based on C10‐BTBTN were measured, and similar results were obtained.

Other comprehensive details of the experimental section can be found in the Supporting Information.

## Conflict of Interest

The authors declare no conflict of interest.

## Supporting information

Supporting Information

## Data Availability

Research data are not shared.
