# Supplementary material for: Thermally Stable Organic Field‐Effect Transistors Based on Asymmetric BTBT Derivatives for High Performance Solar‐Blind Photodetectors
Source: Adv Sci (Weinh). 2022 Feb 19;9(12):2106085. doi: 10.1002/advs.202106085 (PMC9036011; doi:10.1002/advs.202106085)
Supplement: Supplementary file 1 — Supporting Information [file ADVS-9-2106085-s001.pdf]

Supporting Information

Thermally Stable Organic Field-Effect Transistors Based on Asymmetric BTBT Derivatives for High Performance Solar-Blind Photodetectors

Yicai Dong,[1,3] Yanan Sun,[1,3] Jie Liu,[1] Xiaosong Shi,[1,3] Haiyang Li,[1,3] Jing Zhang,[1,3] Chunlei Li,[1,3] Yuanping Yi,[1] Song Mo,[2] Lin Fan[2] and Lang Jiang[1]*

Y. C. Dong, Y. N. Sun, Prof. J. Liu, X. S. Shi, H. Y. Li, J. Zhang, C. L. Li, Prof. Y. P. Yi, Prof. L. Jiang
[1]Beijing National Laboratory for Molecular Sciences, Key Laboratory of Organic Solids, Institute of Chemistry, Chinese Academy of Sciences, Beijing 100190, China.

S. Mo, Prof. L. Fan
[2]Key Laboratory of Science and Technology on High-tech Polymer Materials, Chinese Academy of Sciences, Institute of Chemistry, Chinese Academy of Sciences, Beijing 100190, China.

Y. C. Dong, Y. N. Sun, X. S. Shi, H. Y. Li, J. Zhang, C. L. Li
[3]University of the Chinese Academy of Sciences, Beijing 100049, China.

E-mail: ljiang@iccas.ac.cn

Experimental Section

Materials synthesis and characterization:

General information: All reagents and chemicals were obtained from commercial resources and used without further purification. ^{1}H NMR spectra were recorded on Advance 400 MHz spectrometer in deuterated chloroform with tetramethylsilane (TMS) as internal reference. All chemical shifts were reported relative to TMS at 0.0 ppm. UV-vis spectra were obtained on a Jasco V-570 UV-vis spectrometer with solution concentration of 1×10^{-5} $molL^{-1}$ in dichloromethane. Thin film samples of 30/50 nm on quartz plates were prepared by vacuum evaporation for UV-vis test. CV was run on a CHI660C electrochemistry station in dichloromethane solution using tetrabutylammonium hexafluorophosphate (Bu_4NPF_6) as electrolyte at a scan speed of 100 mVs^{-1}, and glassy carbon was used as the working electrode and Pt wire as the counter electrode. TGA was carried out on a Perkin Elmer TGA7 under nitrogen. DSC curves were obtained by DSC 250 under nitrogen with the heating and cooling rate of 10 $^oCmin^{-1}$.

Synthesis of 2-naphthyl-[1]benzothieno[3,2-b][1]benzothiophene (BTBTN):

BTBT-Br (1a, Scheme S1) were prepared according to the reported literature.[1]

2-naphthyl-[1]benzothieno[3,2-*b*][1]benzothiophene (BTBTN): A mixture of compound 1a (1.6 g, 5 mmol), naphthalene-2-boronic acid (934 mg, 5.44 mmol), K_2CO_3 (2.0 g, 14.8 mmol) and $Pd(PPh_3)_4$ (284 mg, 0.26 mmol) was dissolved in 40 mL oxygen-free toluene under argon. Then 7 mL degassed water was added. The mixture was stirred for 15 h at 80 °C, and then the

mixture was allowed to be cooled to room temperature. Precipitate was obtained after the addition of 20 mL water to the mixture. The precipitate was washed three times with anhydrous ethanol and further purified by physical vapor transport, affording 878 mg (48%) BTBTN as white powder. MS (EI) m/z: 366 (M^+). Elem. anal. calculated for $C_{24}H_{14}S_2$ (%): C: 78.65, H: 3.85. Found: C: 78.54, H: 3.76.

Synthesis of 2-hexyl-7-naphthyl [1]benzothieno[3,2-b][1]benzothiophene (C6-BTBTN):

Compounds of 1b, 2b, and 3b (Scheme S1) were prepared according to the reported literature.[2]

2-hexyl-7-naphthyl [1]benzothieno[3,2-*b*][1]benzothiophene (C6-BTBTN):

A mixture of compound 3b (1.0 g, 2.48 mmol), naphthalene-2-boronic acid (467 mg, 2.72 mmol), K_2CO_3 (1.0 g, 7.4 mmol) and $Pd(PPh_3)_4$ (142 mg, 0.13 mmol) was dissolved in 30 mL oxygen-free toluene under argon. Then 4 mL degassed water was added. The mixture was stirred for 10 h at 80 °C, and then the mixture was allowed to be cooled to room temperature. The grey white precipitate was obtained after the addition of 20 mL water to the mixture. The precipitate was washed three times with anhydrous ethanol and further purified by recrystallization from toluene, affording 647 mg (58%) C6-BTBTN as colourless powder. ^{1}H NMR (400 MHz, $CDCl_3$) δ 8.26 (d, *J* = 1.2 Hz, 1H), 8.15 (s, 1H), 7.97 (s, 1H), 7.96 - 7.92 (m, 2H), 7.91 - 7.87 (m, 1H), 7.83 (ddd, *J* = 8.1, 7.4, 3.3 Hz, 3H), 7.74 (s, 1H), 7.56 - 7.48 (m, 2H), 7.30 (dd, *J* = 8.1, 1.3

Hz, 1H), 2.81 - 2.75 (m, 2H), 1.75 - 1.67 (m, 2H), 1.36 (ddd, *J* = 11.5, 10.2, 5.8 Hz, 6H), 0.90 (t, *J* = 7.0 Hz, 3H). MS (EI) m/z: 450 (M^+). Elem. anal. calculated for $C_{30}H_{26}S_2$ (%): C: 79.95, H: 5.82. Found: C: 79.75, H: 5.76.

Synthesis of 2-octyl-7-naphthyl [1]benzothieno[3,2-b][1]benzothiophene (C8-BTBTN):

As shown in Scheme S1, C8-BTBTN was synthesized by the same synthetic route as C6-BTBTN using octanoyl chloride as starting reagent, and purified by recrystallization from toluene to afford a colourless powder (55%). ^{1}H NMR (400 MHz, $CDCl_3$) δ 8.25 (d, *J* = 1.2 Hz, 1H), 8.15 (s, 1H), 7.97 (s, 1H), 7.96 - 7.91 (m, 2H), 7.91 - 7.87 (m, 1H), 7.83 (ddd, *J* = 8.1, 7.4, 3.4 Hz, 3H), 7.74 (s, 1H), 7.56 - 7.48 (m, 2H), 7.30 (dd, *J* = 8.1, 1.3 Hz, 1H), 2.81 - 2.73 (m, 2H), 1.74 - 1.67 (m, 2H), 1.32 (dd, *J* = 18.3, 10.4 Hz, 10H), 0.89 (t, *J* = 6.9 Hz, 3H). MS (EI) m/z: 478 (M^+). Elem. anal. calculated for $C_{32}H_{30}S_2$ (%): C: 80.29, H: 6.32. Found: C: 80.13, H: 6.24.

Synthesis of 2-decyl-7-naphthyl [1]benzothieno[3,2-b][1]benzothiophene (C10-BTBTN):

C10-BTBTN was synthesized with similar procedure of C6-BTBTN in scheme S1 and the yield is 62%.^{1}H NMR (400 MHz, $CDCl_3$) δ 8.26 (d, *J* = 1.2 Hz, 1H), 8.15 (s, 1H), 7.97 (s, 1H), 7.96 - 7.92 (m, 2H), 7.91 - 7.87 (m, 1H), 7.86 - 7.79 (m, 3H), 7.74 (s, 1H), 7.56 - 7.48 (m, 2H), 7.30 (dd, *J* = 8.2, 1.3 Hz, 1H), 2.81 - 2.73 (m, 2H), 1.70 (dd, *J* = 14.9, 7.3 Hz, 2H), 1.31 (d, *J* = 33.3 Hz, 14H), 0.88

(t, J = 6.9 Hz, 3H). MS (EI) m/z: 506 (M^+). Elem. anal. calculated for $C_{34}H_{34}S_2$ (%): C: 80.58, H: 6.76. Found: C: 80.47, H: 6.86.

2D molecular crystal preparation and device fabrication: High quality ultrathin 2D molecular crystals of C6-BTBTN, C8-BTBTN, and C10-BTBTN were grown on octadecytrichlorosilane (OTS) treated Si/SiO_2 substrates by PVT method under argon atmosphere. In a horizontal tube furnace, the compounds were placed in a quartz boat at the heating zone of 180 °C for 3 h, and then ultrathin single crystals could be obtained at crystallization zone down the argon stream. After that, single crystal transistors with bottom-gate top-contact configuration were fabricated, where gold (~100 nm) source and drain electrodes were fabricated by mechanical transfer. Devices with variable channel length were also fabricated by this method.

Characterization of 2D molecular crystals and measurements of transistors: Optical microscopy images and POM images were recorded by a Leica DM4 M. XRD measurements were performed in reflection mode at 40 kV and 200 mA with Cu Ka radiation using a 2 kW Rigaku D/max-2500 X-ray diffractometer. Thick XRD samples were obtained by increasing temperature and growth time in the PVT process to obtain multi-order diffraction peaks.

AFM and HR-AFM results were obtained by Cypher S (Asylum Research). The electrical characteristics were measured by Agilent B1500A in ambient condition, and corresponding saturation mobility was calculated by using the equation:

$$I_{DS} = \frac{W}{2L} \times C_i \times \mu \times (V_G - V_T)^2 \quad (1)$$

Where W and L are the width and length of channels, respectively, and C_i (10 $nFcm^{-2}$) is the capacitance of the gate-dielectric capacitance per unit area. Device contact resistance R_c were obtained by using TLM method, which were extracted from the transfer curves of the OFETs measured in the linear region at a drain-source voltage (V_{DS}) of -1.0 V. The R_c was calculated by the equation:

$$R_{total}W = R_cW + \frac{L}{\mu C_i(V_G - V_{TH})} \quad (2)$$

Where R_{total} is the total resistance of the OFETs, W is a fixed width and L is channel length. According to the equation, R_c could be obtained from the intercept in the function of width-normalized total device resistance ($R_{total}W$) versus the channel length.

Thin-film transistors fabrication and measurements: Thin-film transistors were fabricated on OTS-treated SiO_2/Si substrates, and 30 nm C10-BTBTN and C8-BTBT thin films were obtained by vacuum evaporation with the speed of 0.1 $Ås^{-1}$. 20 nm gold source and drain electrodes were deposited successively using the shadow masks with width-to-length ratio (W/L, 240 μm/30 μm) of cal. 8/1. Thermal stability measurements were carried out at the heating rate of 0.1°Cs^{-1}.

Photodetectors fabrication and measurements: Photo response characteristics were tested by MPL-N-266nm-20mW laser and light intensity was measured by PM100D optical power meter. The spot of MPL-N-266nm-20mW laser is too small, so the phototransistor matrix was tested by 254 nm commercial hand

lamp. Flexible transistors were fabricated on PI substrates and dielectric were obtained by spin coating PI solution with the speed of 3000 rs^{-1}. 100 nm Al as gate electrode was obtained by vacuum evaporation with the speed of 0.5-4 $Ås^{-1}$.

Theoretical calculation: The neutral ground state Cn-BTBTN (n = 0, 6, 8, 10) molecular structure optimization and frontal orbital distribution were obtained by density functional theory (DFT) at the B3LYP/6-31G** level. Based on the optimized ground-state geometries, the electronic and optical absorption properties were calculated by DFT and time-dependent DFT (TDDFT) at the tuned ωB97X/6-31G** level. All the DFT and TDDFT calculations were carried out using the Gaussian 09 program package. [3]

Based on the XRD data collected, the crystal structure of C10-BTBTN was optimized using the ab initio calculation program CASTEP in Materials Studio. The DFT calculation was performed by generalized gradient approximation (GGA) with the Perdew-Burke-Ernzerhof (PBE) exchange-correlation functional including the Grimme D3 dispersion correction (DFT-D3).[4] The accuracy of the electronic self-consistent field calculation (SCF) is set to 1.0×10^{-6} $eVatom^{-1}$.

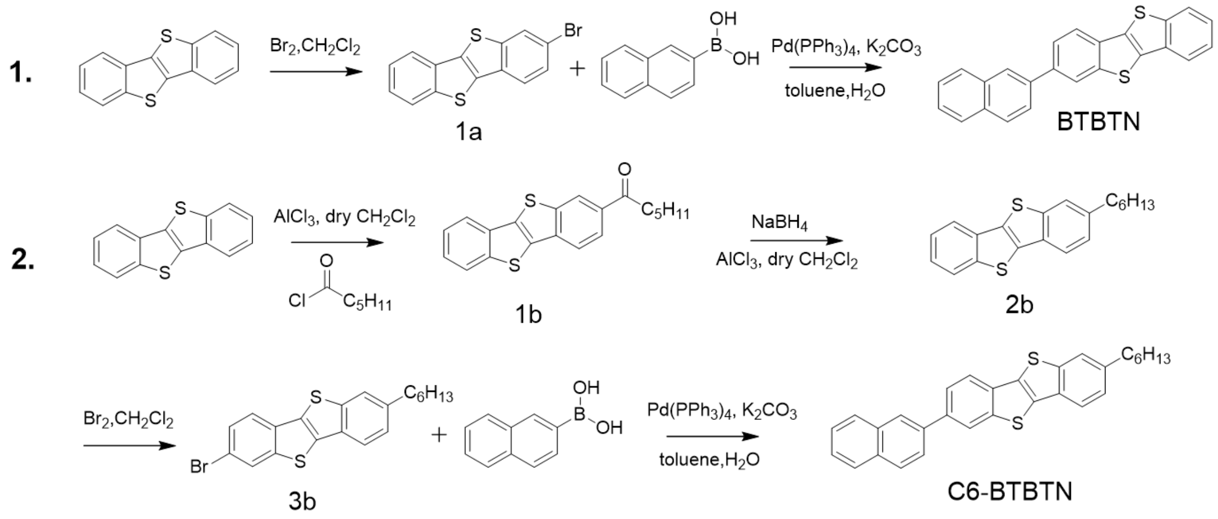

Scheme S1. Synthetic routes of BTBTN and C6-BTBTN.

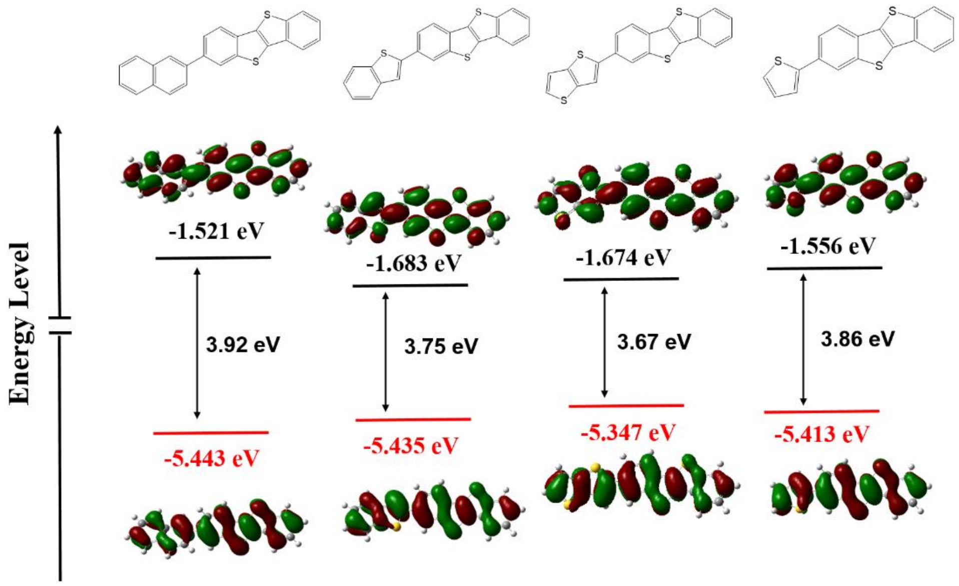

Figure S1. Frontier orbital distributions calculated at the B3LYP/6-31G* level for a series of asymmetric BTBT derivatives with different aromatic substituents at one end (BTBTN, BTBTTB, BTBTTT, BTBTT).

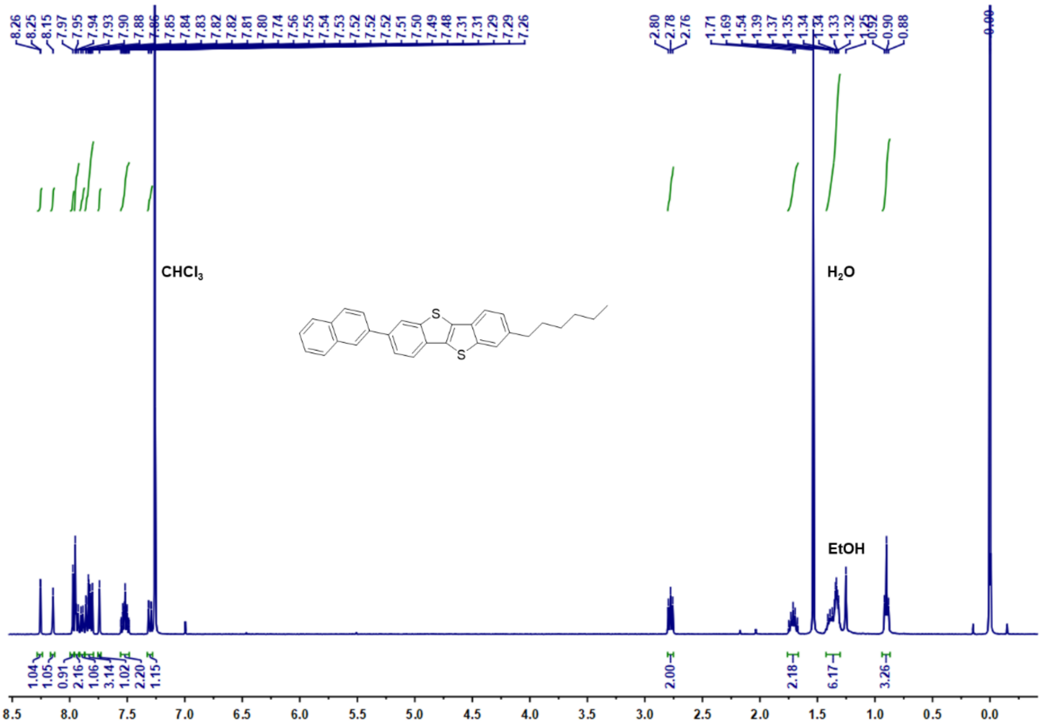
8.26
8.25
8.15
7.97
7.95
7.94
7.93
7.90
7.88
7.86
7.85
7.84
7.83
7.82
7.82
7.81
7.80
7.74
7.56
7.55
7.54
7.53
7.52
7.52
7.52
7.51
7.50
7.49
7.48
7.31
7.31
7.29
7.29
7.26
2.80
2.78
2.76
1.71
1.69
1.54
1.39
1.37
1.35
1.34
1.33
1.32
1.25
0.92
0.90
0.88
0.00
CHCl3
H2O
EtOH
S
S
1.04
1.05
0.91
2.16
1.06
3.14
1.02
2.20
1.15
2.00
2.18
6.17
3.26
8.5 8.0 7.5 7.0 6.5 6.0 5.5 5.0 4.5 4.0 3.5 3.0 2.5 2.0 1.5 1.0 0.5 0.0

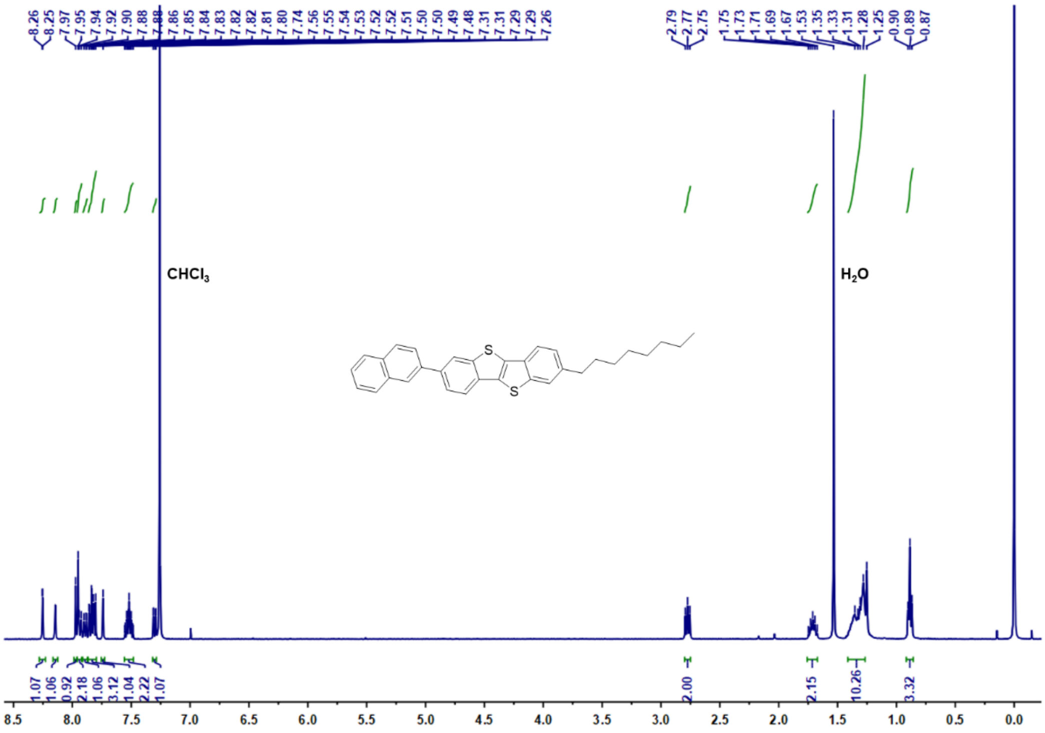
8.26
8.25
7.97
7.95
7.94
7.92
7.90
7.88
7.88
7.86
7.85
7.84
7.83
7.82
7.82
7.81
7.80
7.74
7.56
7.55
7.54
7.53
7.52
7.52
7.51
7.50
7.49
7.48
7.31
7.31
7.29
7.29
7.26
2.79
2.77
2.75
1.75
1.73
1.71
1.69
1.67
1.53
1.35
1.33
1.31
1.28
1.25
0.90
0.89
0.87
CHCl3
H2O
S
S
1.07
1.06
0.92
2.18
1.06
3.12
1.04
2.22
1.07
2.00
2.15
10.26
3.32
8.5 8.0 7.5 7.0 6.5 6.0 5.5 5.0 4.5 4.0 3.5 3.0 2.5 2.0 1.5 1.0 0.5 0.0

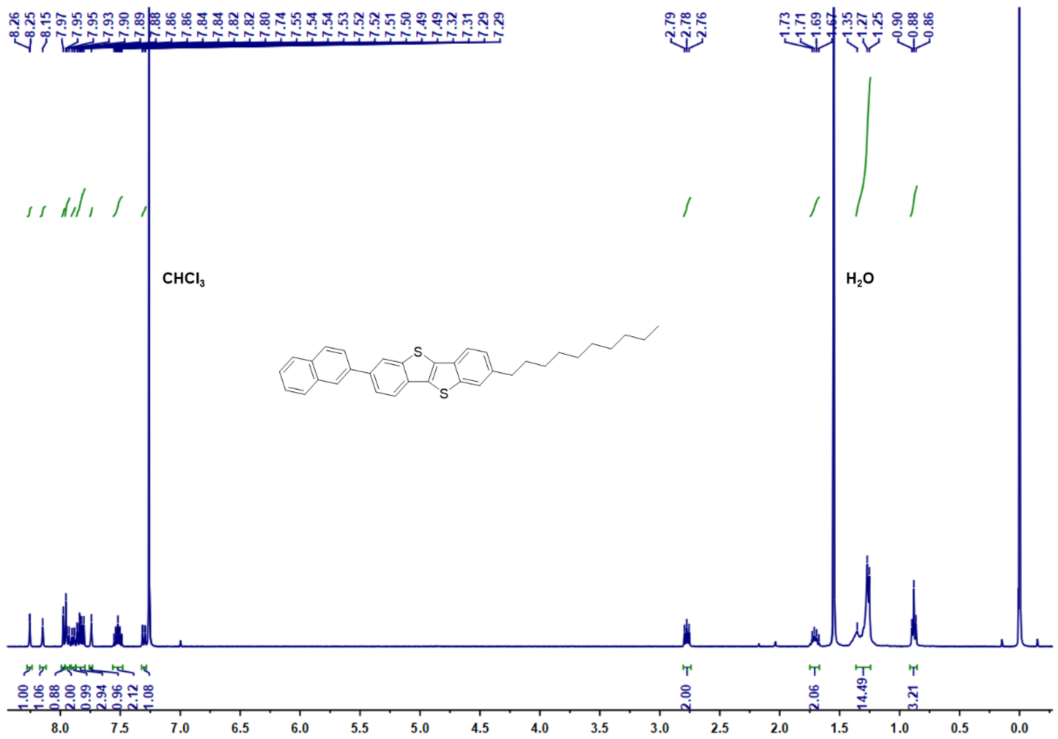

Figure S2. ^{1}H NMR results of Cn-BTBTN (n = 6, 8, 10).

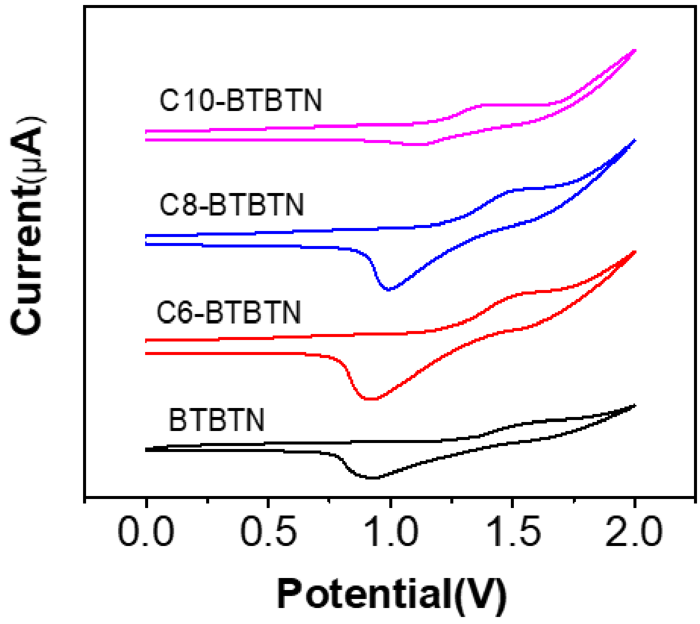

Figure S3. CV curves of Cn-BTBTN (n = 0, 6, 8, 10) in dichloromethane with a concentration of 10^{-3} M at a scan speed of 100 mVs^{-1}.

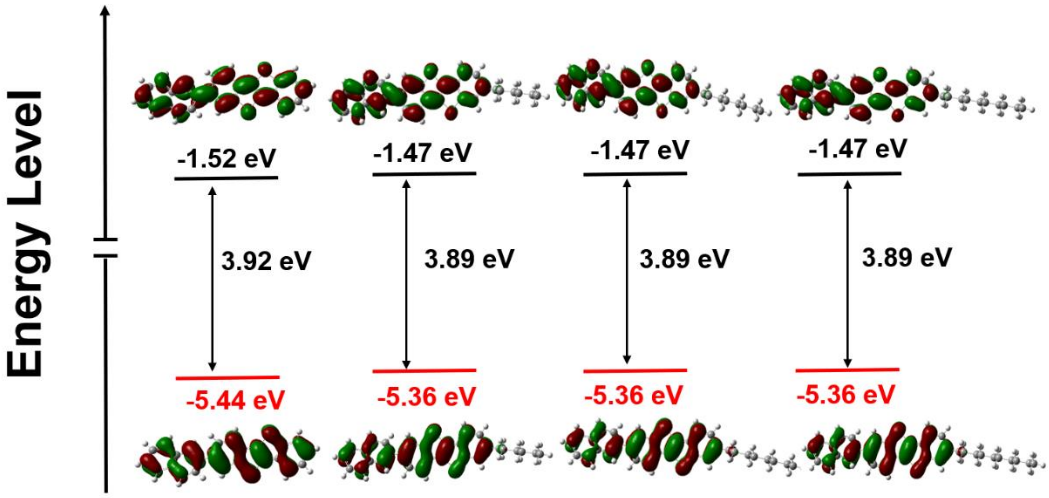

Figure S4. Frontier orbital distributions for Cn-BTBTN (n = 0, 6, 8, 10) molecules calculated at the B3LYP/6-31G* level.

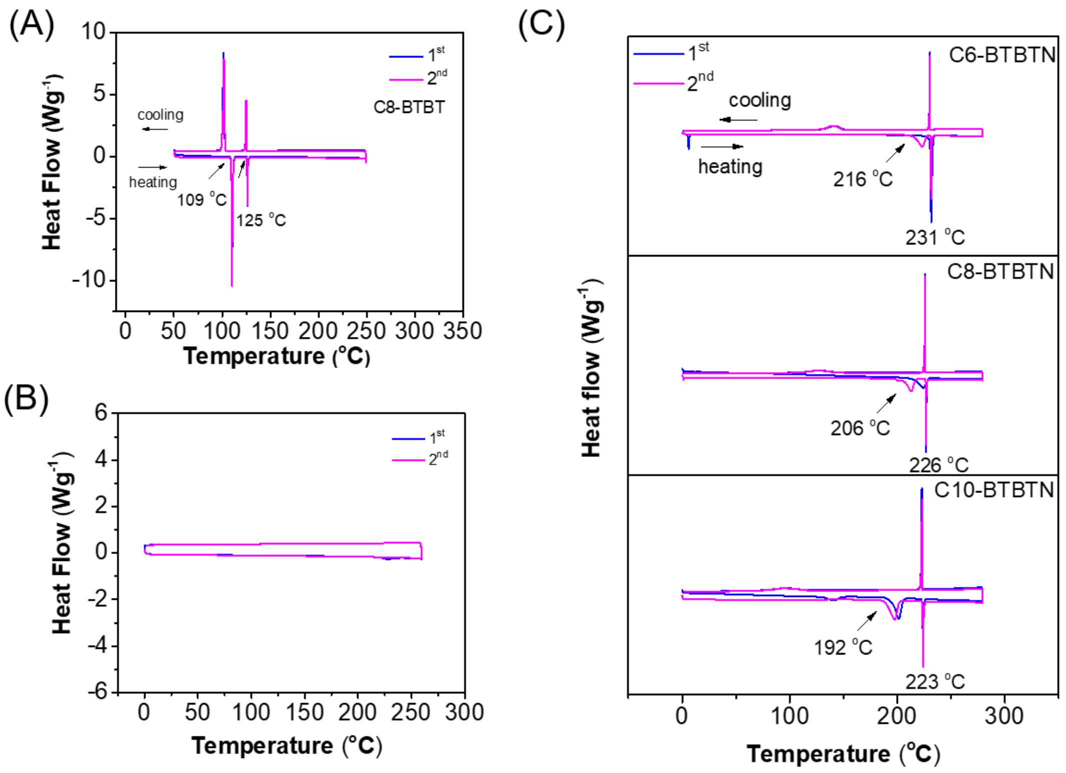

Figure S5. (A) DSC curves of C8-BTBT molecule. (B, C) DSC curves of Cn-BTBTN (n = 0, 6, 8, 10) molecules. Blue curves show the results for first scans at 10 °C min^{-1}, and red curves show the results for second scans at 10 °C min^{-1}.

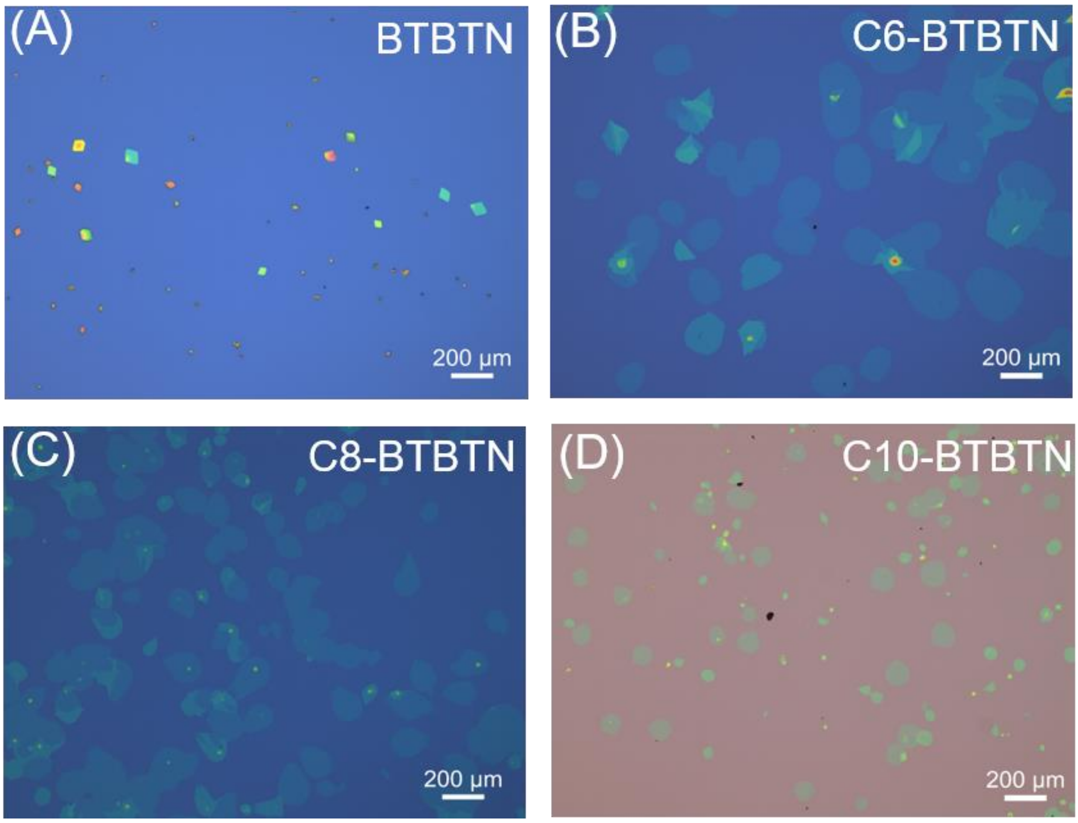

Figure S6. Optical microscopy images for (A) BTBTN, (B) C6-BTBTN, (C) C8-BTBTN and (D) C10-BTBTN single crystals.

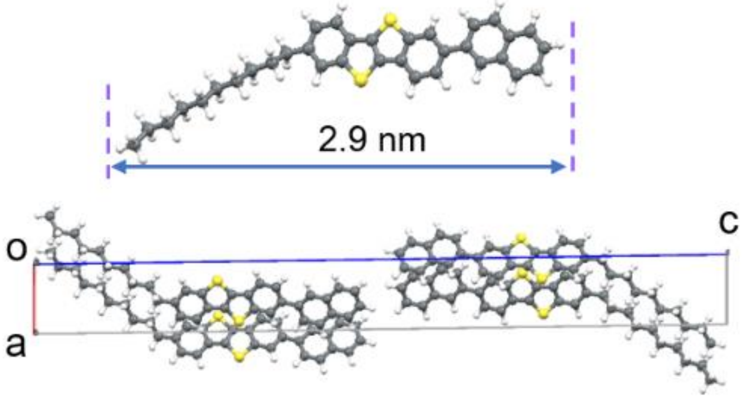

Figure S7. Unit-cell packing diagrams of C10-BTBTN: molecular length and projection of a-c plane. C10-BTBTN crystal structure based on partially collected single crystal results, and packing structure in solid state was preasented. The molucular length of C10-BTBTN was 2.9 nm and the crystals adopted layer-by-layer arrangement with the molecular long axes aligned roughly perpendicular to other layers. Attempts for other compounds met with limitted results, and we assume that similar packing motifs might be adopted for C6 and C8-BTBTN.

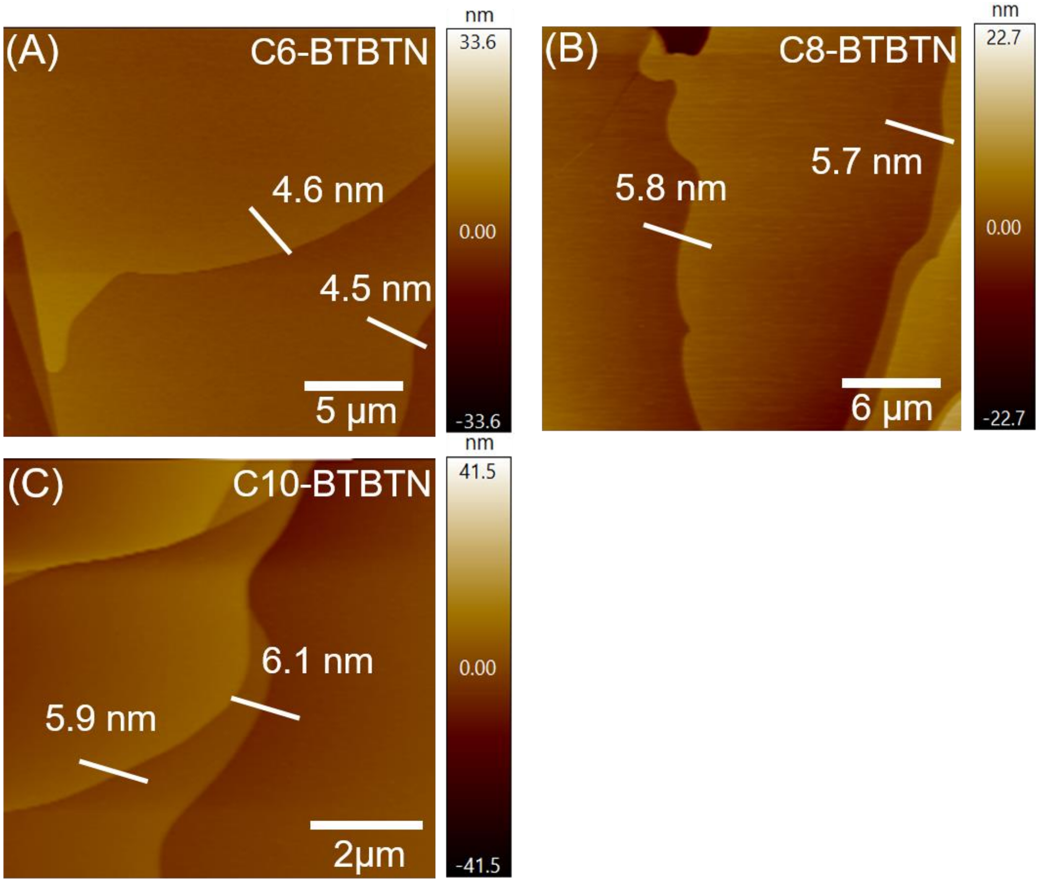

Figure S8. (A-C) AFM images of multilayer molecular crystals of Cn-BTBTN (n = 6, 8, 10).

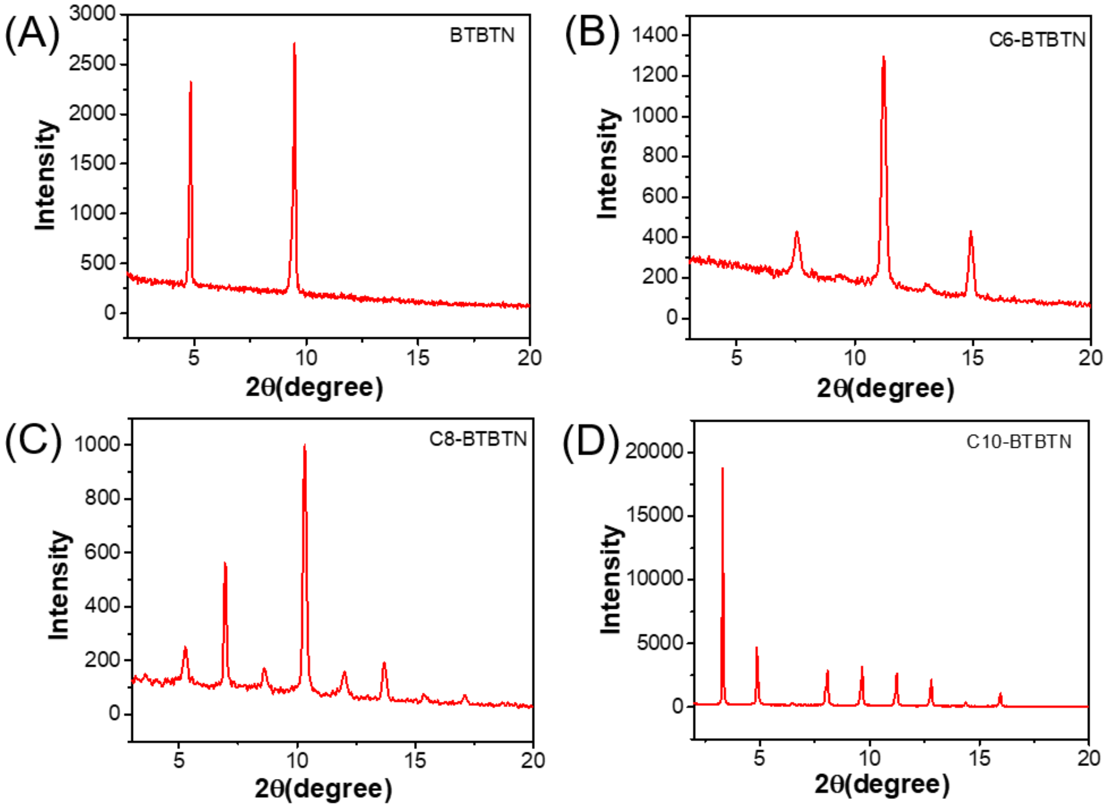

Figure S9. (A-D) Out of plane XRD results of Cn-BTBTN single crystals (n = 0, 6, 8, 10).

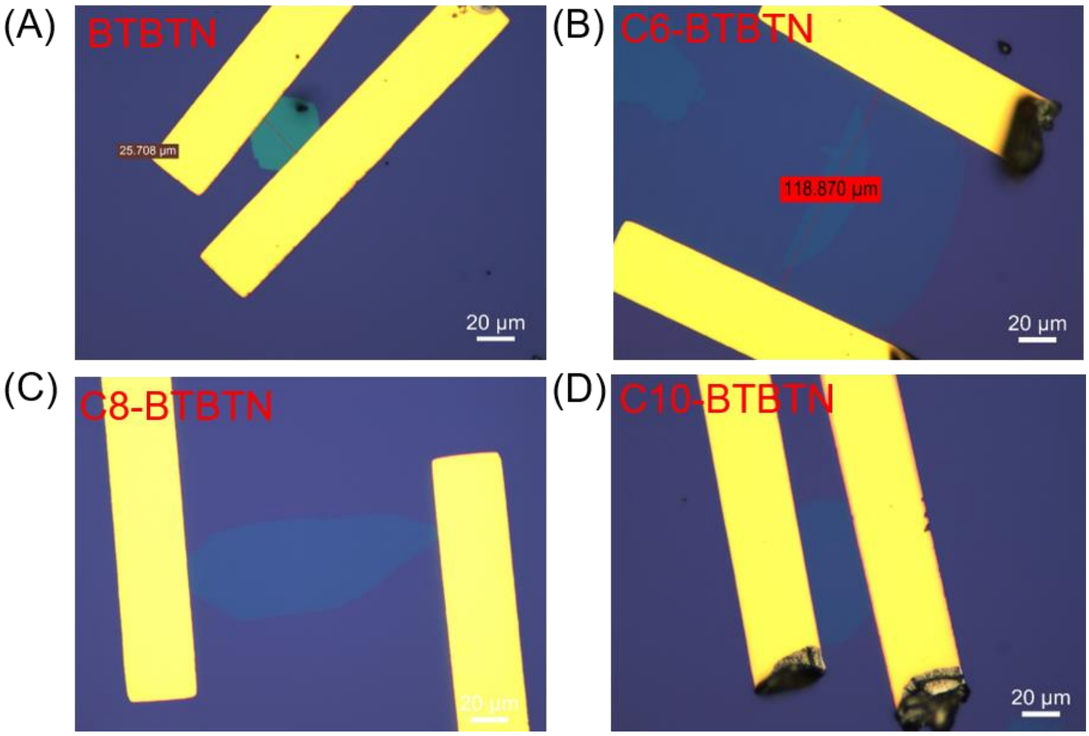

Figure S10. Optical images of BTBTN, C6-BTBTN, C8-BTBTN, and C10-BTBTN devices, respectively.

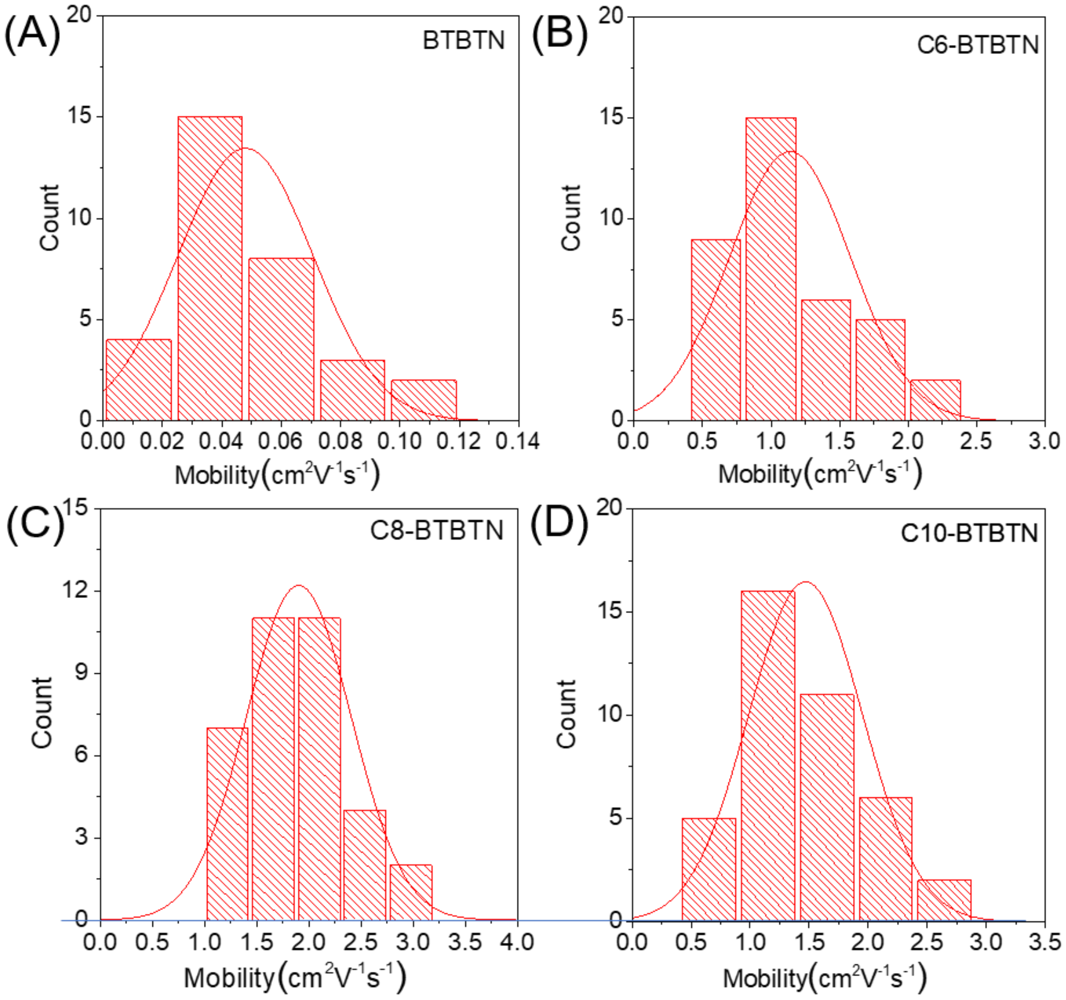

Figure S11. Mobility distribution of BTBTN, C6-BTBTN, C8-BTBTN and C10-BTBTN single-crystal devices, respectively.

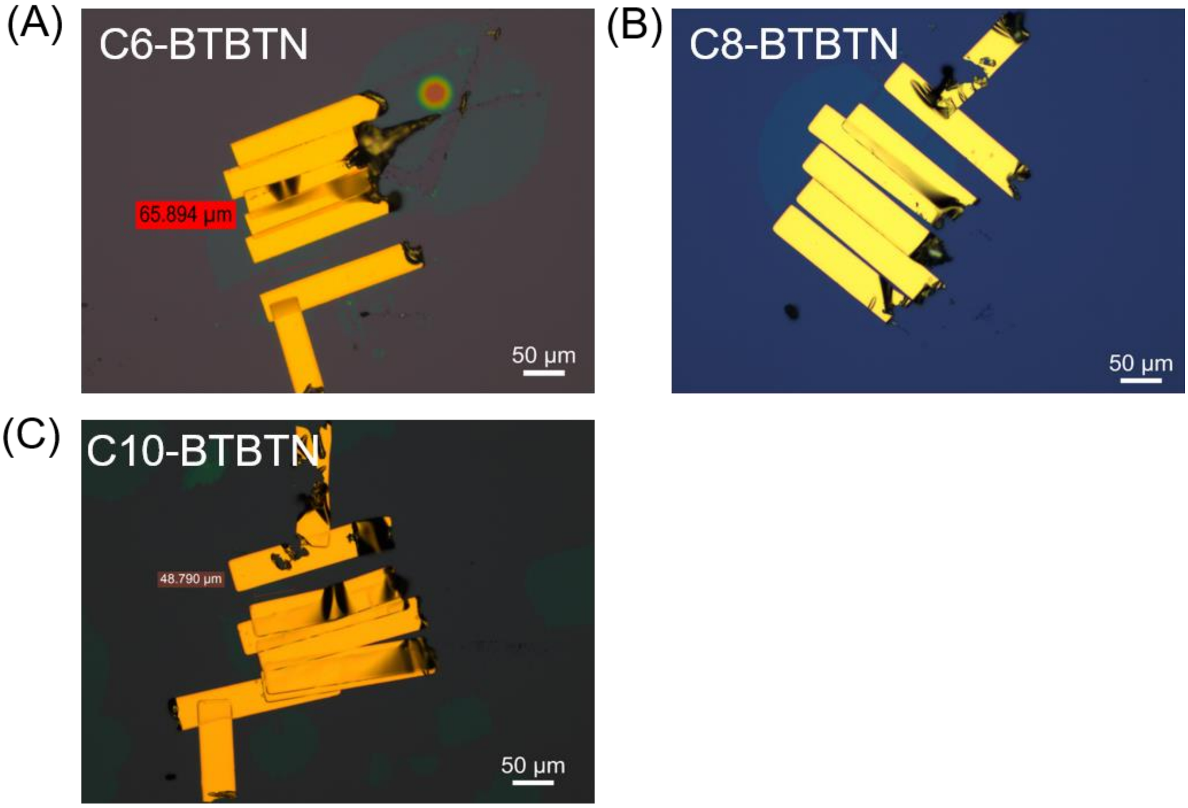

Figure S12. OM images of of BTBTN, C6-BTBTN, C8-BTBTN, and C10-BTBTN devices, respectively.

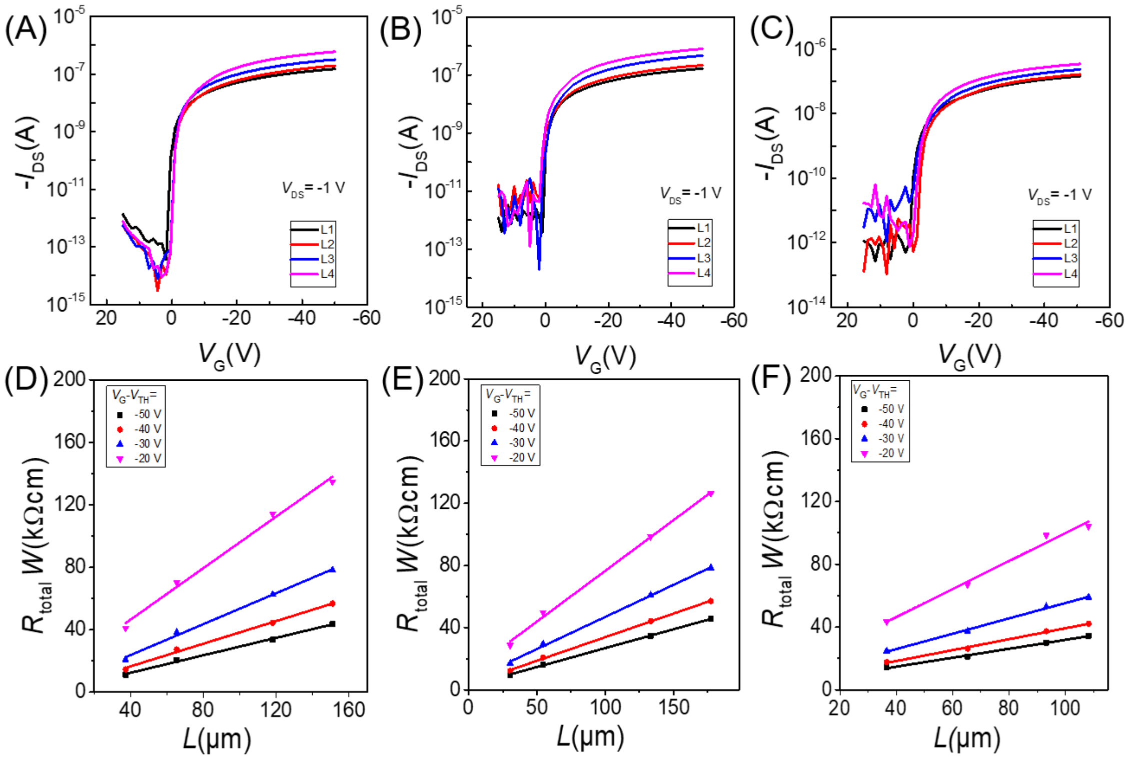

Figure S13. Transfer curves measured in the linear regime of bilayer devices of (A) C6-BTBTN, (B) C8-BTBTN and (C) C10-BTBTN, respectively. Total device resistance calculated using TLM and plotted as a function of the channel length of the transistors for various gate over drive voltages ($V_G - V_{TH}$) for the devices of (D) C6-BTBTN, (E) C8-BTBTN, and (F) C10-BTBTN, respectively. TLM measurements were conducted on bilayer single crystals OFETs to estimate the contact resistance. According to the equation (2) shown in experimental section, the estimated width-normalized $R_{total}\cdot W$ for C6-, C8-, C10-BTBTN are 1.22 kΩcm, 2.58 kΩcm, and 3.39 kΩcm, respectively.

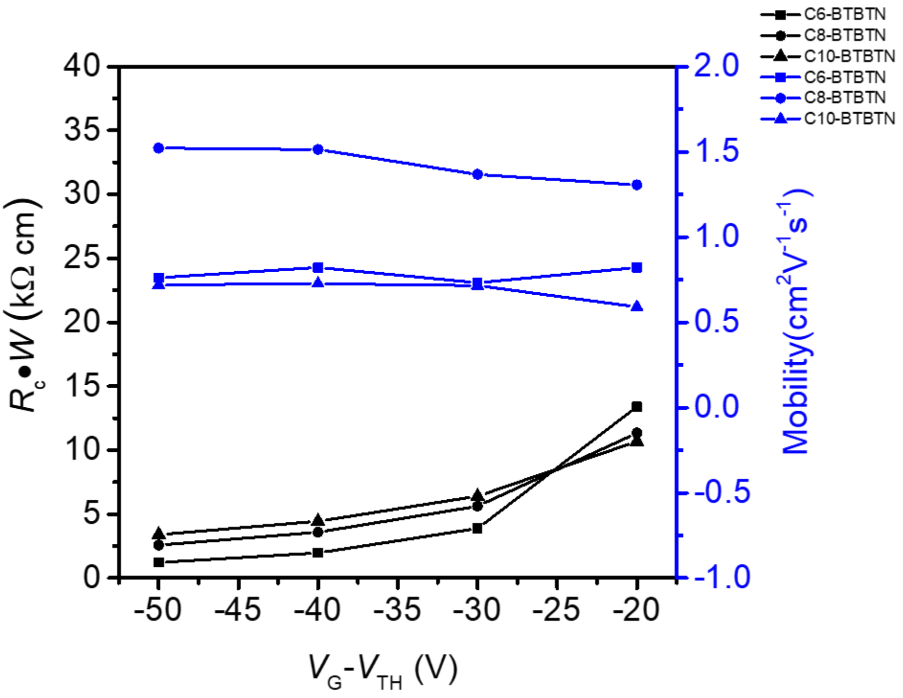

Figure S14. Calculated contact resistance (black squares) and intrinsic mobility (blue squares) at various gate voltages ($V_G - V_{TH}$) for the bilayer devices for C6-BTBTN, C8-BTBTN and C10-BTBTN, respectively.

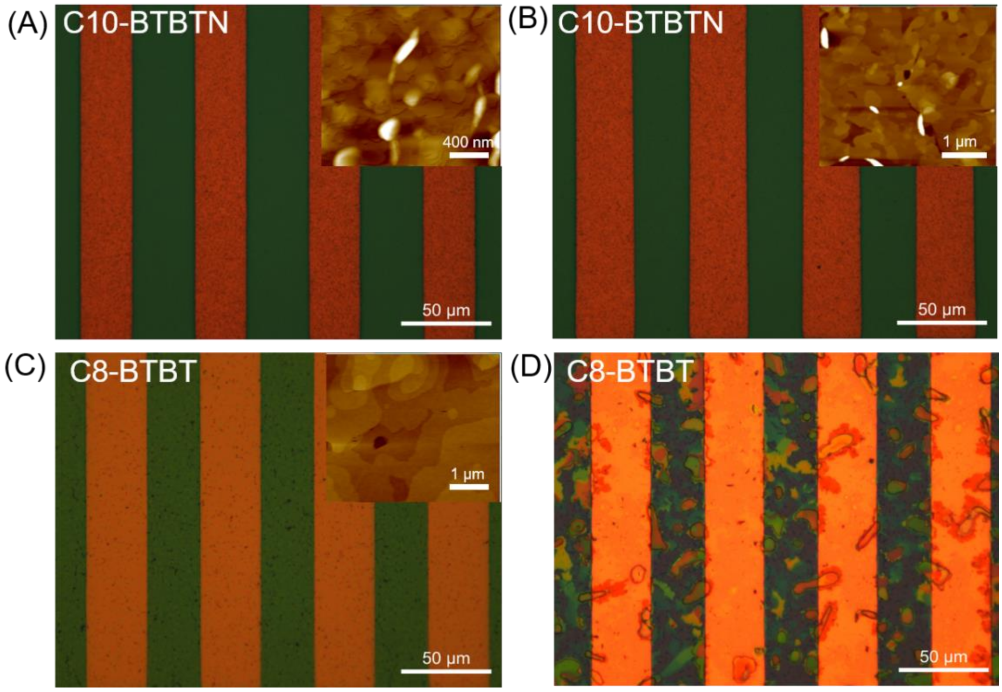

Figure S15. OM images of C10-BTBTN and C8-BTBT TFTs without heating (A,C) and after 120 °C heating (B,D).

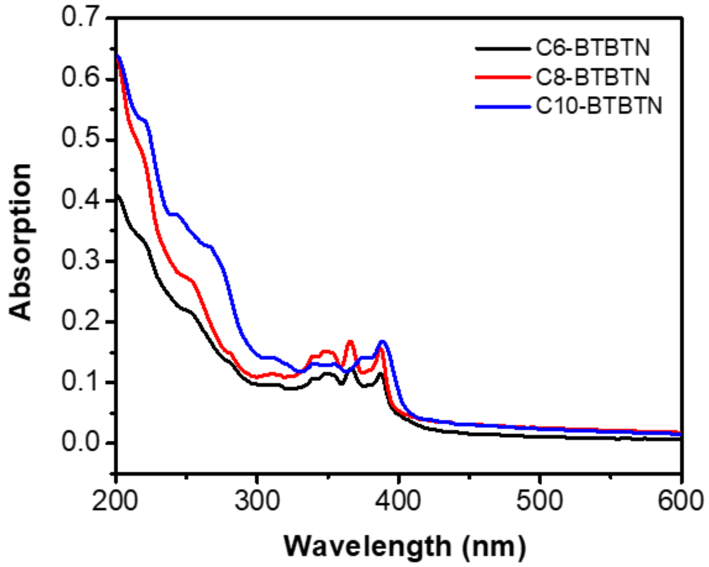

Figure S16. UV-vis absorption spectra of 50 nm Cn-BTBTN (n = 6, 8, 10) thin films on quartz.

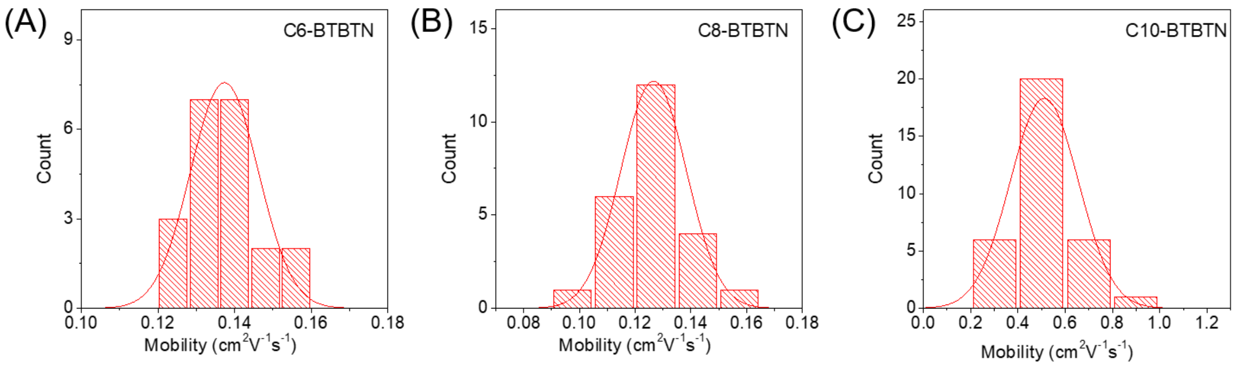

Figure S17. (A-C) Mobility distribution of C6-BTBTN, C8-BTBTN, and C10-BTBTN thin-film devices, respectively.

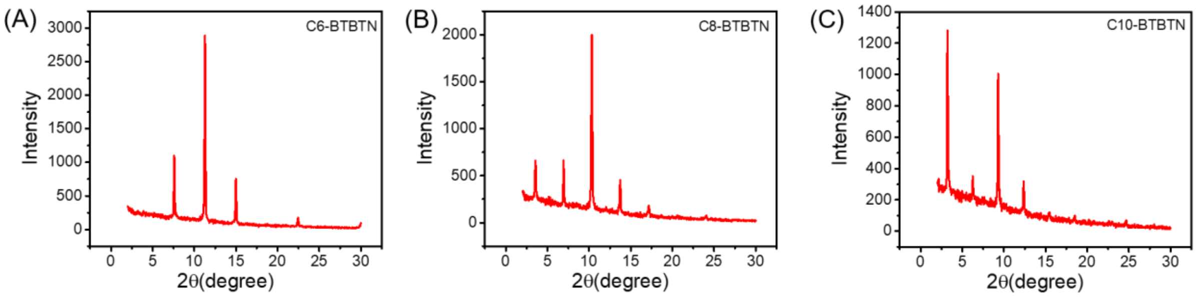

Figure S18. (A-C) Out of plane XRD results of Cn-BTBTN thin films (n = 6, 8, 10).

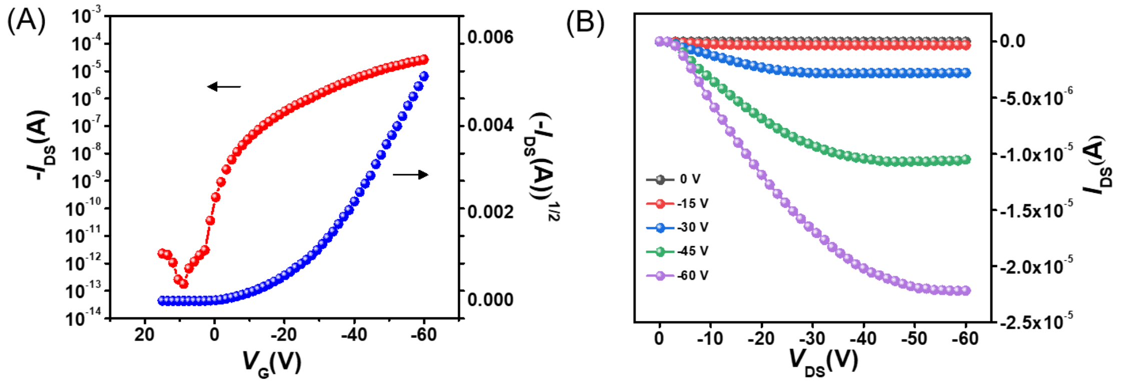

Figure S19. (A)Typical transfer and (B) output characteristics on OTS-treated SiO_2 substrate.

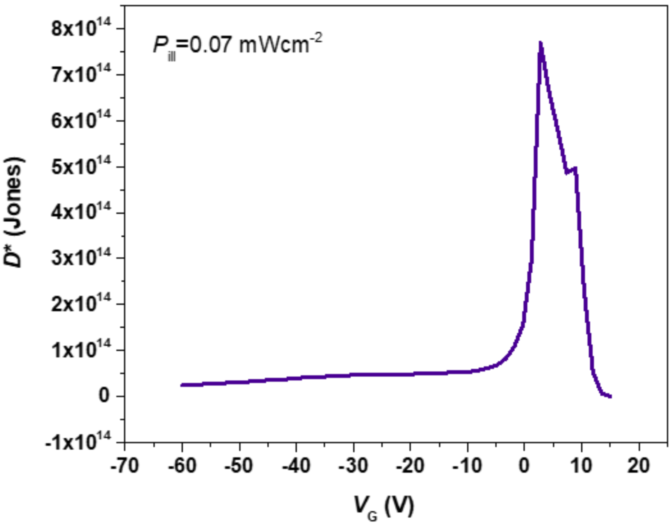

Figure S20. D^* at different V_G under wavelength illumination at 0.07 mWcm^{-2}.

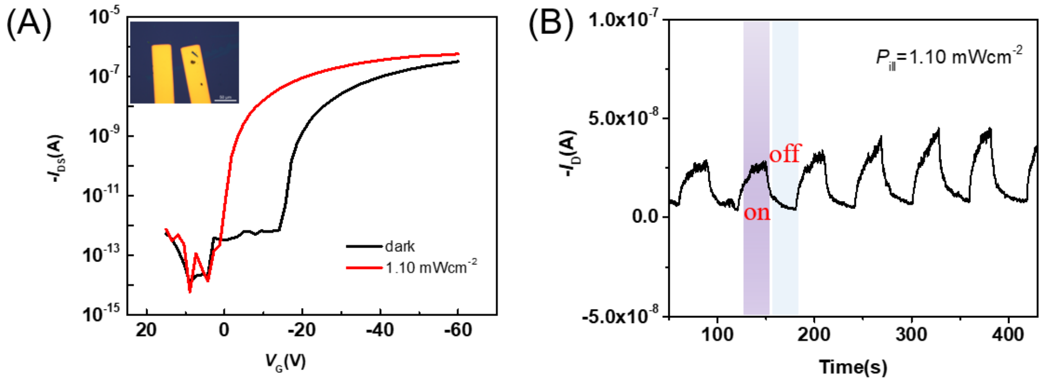

Figure S21. (A) Transfer characteristics of C10-BTBTN single crystal-based photodetectors measured in dark and under 266 nm illumination. (B) Photoswitching characteristics of single crystal-based photodetectors at V_{DS} = −60V and V_G = -10V.

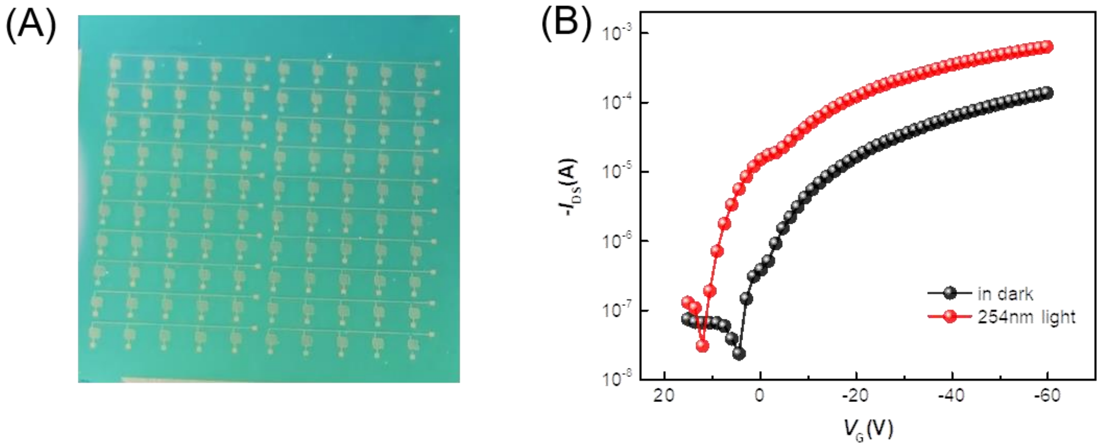

Figure S22. (A) Image of the phototransistor array. (B) Transfer curves of C10-BTBTN-based phototransistor measured in dark and under 254 nm illumination of commercial hand lamp.

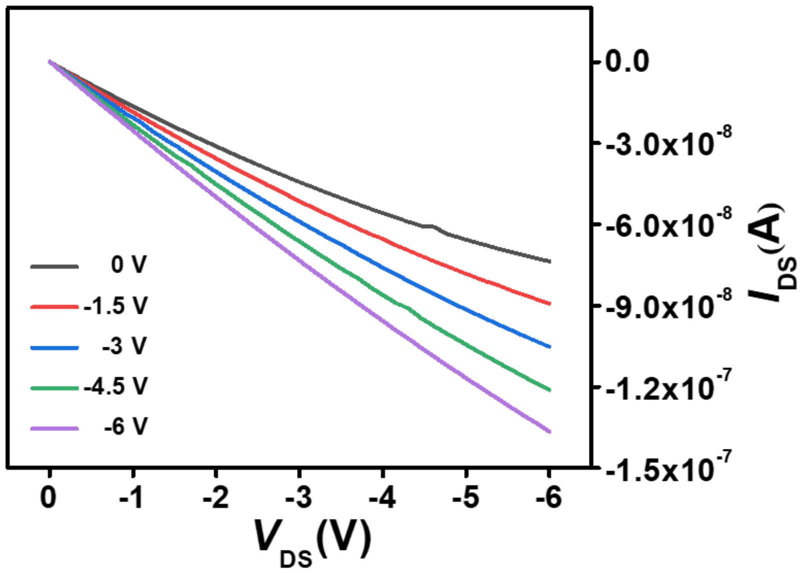

Figure S23. Output characteristics of C10-BTBTN flexible phototransistors at 0.07 $mWcm^{-2}$ illumination intensity.

Table S1. Excitation wavelengths (λ, nm), oscillator strengths (f), and main electronic transitions and corresponding weights for C10-BTBTN and C8-BTBT.

System	Excitation	λ (nm)	f	Transition (weight)
C10-BTBTN	S1	305	1.224	HOMO-LUMO (85%)
	S7	235	0.838	HOMO-LUMO+4 (25%)<br>HOMO-LUMO+2 (17%)<br>HOMO-1-LUMO+1(9.8%)
C8-BTBT	S1	289	0.610	HOMO-LUMO (92%)<br>HOMO-1-LUMO+2 (2%)
	S2	273	0.275	HOMO-1-LUMO (78%)<br>HOMO-LUMO+2 (12%)
	S5	229	0.466	HOMO-1-LUMO+2 (59%)<br>HOMO-2-LUMO+1 (11%)<br>HOMO-4-LUMO (12%)
	S9	209	0.511	HOMO-LUMO+2 (69%)<br>HOMO-1-LUMO (16%)<br>HOMO-4-LUMO (5%)

Table S2. The average mobility and absorption intensity of 50 nm Cn-BTBTN (n = 6, 8, 10) films.

Thin films	Average mobility ($cm^2V^{-1}s^{-1}$)	Absorption intensity (at 266nm)
C6-BTBTN	0.14 ± 0.01	0.171
C8-BTBTN	0.13 ± 0.01	0.202
C10-BTBTN	0.51 ± 0.14	0.325

References

[1] A. Sanzone, S. Mattiello, G. M. Garavaglia, A. M. Calascibetta, C. Ceriani, M. Sassi, L. Beverina, *Green Chem.* **2019**, 21, 4400.

[2] S. Inoue, H. Minemawari, J. Tsutsumi, M. Chikamatsu, T. Yamada, S. Horiuchi, M. Tanaka, R. Kumai, M. Yoneya, T. Hasegawa, *Chem. Mater.* **2015**, 27, 3809.

[3] O. V. Borshchev, A. S. Sizov, E. V. Agina, A. A. Bessonov, S. A. Ponomarenko, *Chem. Commun (Camb).* **2017**, 53, 885.

[4] Y. Zeng, R. Duan, Y. Guo, G. Han, Q. Li, Y. Yi, *Chin. Chem. Lett.* **2019**, 30, 211.
